# Dietary betaine prevents obesity through gut microbiota-drived microRNA-378a family

**DOI:** 10.1080/19490976.2020.1862612

**Published:** 2021-02-08

**Authors:** Jingjing Du, Peiwen Zhang, Jiang Luo, Linyuan Shen, Shunhua Zhang, Hao Gu, Jin He, Linghui Wang, Xue Zhao, Mailing Gan, Liu Yang, Lili Niu, Ye Zhao, Qianzi Tang, Guoqing Tang, Dongmei Jiang, Yanzhi Jiang, Mingzhou Li, Anan Jiang, Long Jin, Jideng Ma, Surong Shuai, Lin Bai, Jinyong Wang, Bo Zeng, De Wu, Xuewei Li, Li Zhu

**Affiliations:** a College of Animal Science and Technology, Sichuan Agricultural University, Chengdu, China; b Farm Animal Genetic Resource Exploration and Innovation Key Laboratory of Sichuan Province, Sichuan Agricultural University, Chengdu, China; c College of Life and Biology Science, Sichuan Agricultural University, Chengdu, China; d Chongqing Academy of Animal Science, Rongchang, China; e Institute of Animal Nutrition, Sichuan Agricultural University, Chengdu, China

**Keywords:** Betaine, obesity, gut microbiota, SCFA, microRNA

## Abstract

Betaine is a natural compound present in commonly consumed foods and may have a potential role in the regulation of glucose and lipids metabolism. However, the underlying molecular mechanism of its action remains largely unknown. Here, we show that supplementation with betaine contributes to improved high-fat diet (HFD)-induced gut microbiota dysbiosis and increases anti-obesity strains such as *Akkermansia muciniphila, Lactobacillus*, and *Bifidobacterium*. In mice lacking gut microbiota, the functional role of betaine in preventing HFD-induced obesity, metabolic syndrome, and inactivation of brown adipose tissues are significantly reduced. *Akkermansia muciniphila* is an important regulator of betaine in improving microbiome ecology and increasing strains that produce short-chain fatty acids (SCFAs). Increasing two main members of SCFAs including acetate and butyrate can significantly regulate the levels of DNA methylation at host miR-378a promoter, thus preventing the development of obesity and glucose intolerance. However, these beneficial effects are partially abolished by Yin yang (YY1), a common target gene of the miR-378a family. Taken together, our findings demonstrate that betaine can improve obesity and associated MS via the gut microbiota-derived miR-378a/YY1 regulatory axis, and reveal a novel mechanism by which gut microbiota improve host health.

## Introduction

Obesity and increased adiposity are characterized by a high body mass index (BMI) and excess fat accumulation in adipose or non-adipose tissues, and are the main causative factors of metabolic syndrome (MS).^1^ Compared with lean individuals, adipose tissues in obese individuals secrete high levels of pro-inflammatory cytokines such as TNF-α, IL-1β, and IL-6, induce systemic inflammation and insulin resistance,^2^ produce large amounts of carcinogenic factors, and increase the risk of certain types of cancer such as melanoma, colon cancer, and liver cancer.^3^ In addition, current evidence suggests that obesity or increased adiposity can lead to an increase in chronic diseases such as nonalcoholic fatty liver disease (NAFLD), cardiovascular diseases, and type 2 diabetes mellitus (T2DM), thus reducing the quality of life and longevity.^4^ Indeed, obesity is a major public health problem worldwide and is replacing undernutrition and infectious diseases as the most significant contributor to ill health. In this respect, the prevalence of obesity and overweightness has doubled in more than 70 countries and has continuously increased in many other countries since the 1980s.^5^ Globally, 107.7 million children and 603.7 million adults were obese in 2015. Furthermore, approximately 3.4 million deaths are caused by obesity-related diseases each year.^6^ Therefore, finding effective therapies for obesity is critical.

The causes of obesity and overweightness are complex and may involve genetic, nutritional, and environmental factors.^4^ The gastrointestinal tract (GIT) is one of the largest interfaces between the outside world and the internal environment in humans and animals. As an indispensable natural process for health benefits, a complex microbial consortium begins immediately at birth in the gastrointestinal tract of humans and animals. These microbial communities can colonize the gut of individuals in subsequent generations.^7,8^ Previous studies have demonstrated that maintaining a mutualistic relationship with gut microbiota is critical for human and animal health because the microbiota not only modulate host physiological processes in the GIT but also help regulate extra-intestinal organs, such as the liver and brain, and the immune system.^9–11^ Obesity and MS are often associated with gut bacterial dysbiosis, with recent evidence revealing a significant difference in the gut microbiota between lean and obese individuals.^12–14^ In addition to fecal microbiota transplants (FMT) from healthy to diseased individuals, prebiotics obtained from fruits and vegetables can regulate host lipid metabolism and glucose homeostasis by reversing gut dysbiosis in obese individuals.^14^

Betaine, also named *N*-trimethylglycine, is a natural substance commonly found in shellfish, wheat, beets, and spinach. Betaine deficiency is associated with MS, including lipid disorders, diabetes, and vascular diseases.^15,16^ However, details of the effect of this substance on obesity and MS and its underlying molecular mechanism remain unknown. In this study, we investigated the effect of betaine on preventing obesity and related metabolic disorders and its underlying mechanisms in mice. Our results suggest that betaine can reduce obesity and associated MS, induce iWAT browning and BAT activation. The mechanism of betaine action may involve gut microbiota-derived miR-378a/*YY1* regulatory axis.

## Materials and methods

### Betaine, butyrate, acetate, and antibiotic treatments

All animal work was performed in accordance with procedures approved by the Animal Care and Ethics Committee at Sichuan Agricultural University. Six-week-old Kunming female mice, purchased from Chengdu Dossy Experimental Animals Co., Ltd (Chengdu, China), were given free access to food and water. After a 1-week adaptation period, mice with similar body weight were housed under controlled conditions (temperature of 25 ± 2°C and a 12–12 h light–dark cycle). Subsequently, the mice were randomly assigned into four groups and were fed with (1) normal chow (Chow; 13.2% fat, 23.2% protein, 63.6% carbohydrate by energy); (2) a high fat diet (HFD; 40.4% fat, 14.6% protein, 45.2% carbohydrate by energy); (3) Chow and 1% betaine (weight/volume; purity ≥98%; B2629, Sigma-Aldrich, St. Louis, MO, USA; Chow+B); and (4) HFD and 1% betaine (HFD+B) for 23 weeks. All feeds were purchased from Chengdu Dossy Experimental Animals Co., Ltd.

To evaluate the effect of butyrate and acetate on obesity and MS, 7-week-old mice were fed with (1) HFD +1% sodium butyrate (purity ≥98.5%; D0004-25 G, BOSF, Taiwan, China), (2) HFD +150 mM sodium acetate (purity ≥99%; S2889-1 KG, Sigma) in water for 60 or 45 days, respectively. In addition, to determine whether betaine improves obesity along with gut microbiota, mice lacking gut microbiota were further fed with HFD and 1% betaine for 45 days. Gut microbiota-depleted mice were generated by an antibiotic cocktail (Abx).^17^ Briefly, 8-week-old Kunming mice were initially supplemented with a 200 μL combination of four nonabsorbable antibiotics including ampicillin, neomycin, metronidazole, and vancomycin (Sangon Biotech, China) via oral gavage daily (10 mg of each antibiotic per mice per day). After 10 days, the oral gavage was changed to ad libitum administration in drinking water (1 g/L ampicillin, 1 g/L neomycin, 1 g/L metronidazole, and 500 mg/L vancomycin) for indicated durations.

### Cell culture and transfection

Mouse 3T3-L1 cells and HeLa cells (Stem Cell Bank, Chinese Academy of Science) were cultured in Dulbecco’s modified Eagle’s medium (DMEM, Gibco, Carlsbad, CA, USA) containing 10% fetal bovine serum (FBS) at 5% CO2 and 37°C. In order to alter the expression levels of miR-378a-3p, miR-378a-5p, Dnmt1, Dnmt3a or Dnmt3b in 3T3-L1 cells, miR-378a-3p mimics, miR-378a-3p inhibitors, miR-378a-5p mimics, miR-378a-5p inhibitors, siRNA of Dnmt1, Dnmt3a or Dnmt3b, both mimics and inhibitors negative controls, siRNA negative controls (all designed and purchased from RIBOBIO, China) were transfected into cells using Lipofectamine3000 (Invitrogen, Carlsbad, CA, USA), following the manufacturer’s recommended protocol. To remove DNA methylation *in vitro*, 70–80% confluent 3T3-L1 cells were treated with 5-Aza-2 deoxycytidine (5-Aza-dC, 189825, Sigma) at a final concentration of 4 μM for 48 h.

### Fecal microbiota and A. muciniphila supplementation

Collection of fecal samples and fecal microbiota transplants (FMT) was performed according to previously established protocols.^14^ Briefly, donor mice were fed with HFD or HFD +1% betaine for 23 weeks (n = 6 per group). After one week of feeding, fresh fecal samples were collected daily from donor mice under a laminar flow hood in sterile conditions. Stools from donor mice of each diet group were pooled, and 100 mg was resuspended in 1 ml of sterile saline. The solution was vigorously mixed for 10 s using a benchtop vortex and centrifuged at 800 *g* for 3 min. The supernatant was then collected and used as FMT material. Fresh fecal material was prepared on the same day of FMT within 10 min before oral gavage to prevent changes in bacterial composition. The 20-week diet induced-obese mice were selected as recipients, divided into two groups (n = 8–10 per group), and inoculated daily with the fecal material (100 μL per mice) by oral gavage for 9 weeks and were humanely killed for subsequent analysis.

To assess the effect of *Akkermansia muciniphila* (*A. muciniphila*; DSM22959) on obesity and MS, *A. muciniphila* was first cultured anaerobically in a mucin-based medium as previously described.^18^ Briefly, the cultures were washed and concentrated in anaerobic phosphate-buffered saline (PBS; SH30256.01, Hyclone, USA) containing 25% (v/v) glycerol to a final concentration of 1.10^10^ colony-forming units (CFU)/mL under strict anaerobic conditions for 72 h. Cultures were immediately frozen and stored at −80°C. A representative glycerol stock was thawed under anaerobic conditions to determine bacterial numbers by plate counting using mucin media containing 1% agarose (agar noble; Difco). Before oral gavage, glycerol stocks were thawed under anaerobic conditions and diluted with anaerobic PBS to a final concentration of 2 × 10^8^ CFU/150 μL and 2.5% glycerol. As recipients, 6-week-old mutated leptin receptor Lepr^db/db^ or C57BL/6 recipient mice that were purchased from GemPharmatech Co., Ltd (Nanjing, China) were inoculated with PBS or the above-described cultures containing *A. muciniphila* (100 μL per mice) by oral gavage daily under Chow conditions. After supplementation for 8 weeks, the Lep^db/db^ recipient mice were humanely killed for subsequent analysis. The C57BL/6 recipient mice supplemented with *A. muciniphila* for eight weeks were further fed with 5-week HFD for the indicated analysis.

### Systemic administration of plasmid DNA in vivo

The hydrodynamics-based procedure involving the systemic administration of naked DNA was performed as described previously.^19^ Briefly, the mouse protein-coding sequence of Yin Yang 1 (*p-YY1*) and mmu-miR-378a precursor sequence (*miR-378a)* were respectively amplified and cloned into pcDNA3.1+ and Plko.1-EGFP-PURO vectors by TsingKe Biotech Co., Ltd (Chengdu, China). Purified 700–900 ng/mL *p*-YY1, premiR-378a, pcDNA3.1(+) empty plasmids or Plko.1-EGFP-PURO empty plasmids DNA in 100 µL saline were injected intraperitoneally into 6-week-old female C57BL/6 mice under HFD conditions, respectively, for 45 days. Injection was performed every three days to confirm high efficiency transgene expression *in vivo*.

### Physiological and biochemical analyses

For serum isolation, blood samples were collected at 4°C from fasted mice, separated by centrifugation at 3000 *g* for 15 min at 4°C and then stored at – 20°C. Total serum triglyceride (TG), total cholesterol (TC), high-density lipoprotein cholesterol (HDL), low-density lipoprotein cholesterol (LDL), aspartate aminotransferase (AST) and alanine aminotransferase (ALT) were measured by Lilai Biotechnology Co., Ltd. The composition of short-chain fatty acids (SCFAs) was determined by Beijing Masspeaks Technology Co., Ltd (Beijing, China) using gas chromatograph-mass spectrometry (GC-MS 7890B-5977A). TG concentrations in tissues and feces were measured using a triglyceride assay kit (Nanjing Jiancheng Bioengineering Institute, A110-1-1).

For the glucose tolerance test (GTT), overnight-fasted mice were injected intraperitoneally with 2 mg glucose/g body weight, following which the levels blood glucose obtained from the tail vein were measured using a glucometer (06583261001; Roche, Germany). In the intraperitoneal insulin tolerance test (ITT), mice fasted for 6 h were given intraperitoneal injections of 0.5 mU insulin/g body weight, and blood glucose levels were measured at 0, 15, 30, 60, and 90 min post injection.

### Detection of DNA synthesis using EdU in vivo

To label the nuclei of dividing adipocytes, experimental mice were intraperitoneally injected on the first or fourth day with 5-ethynyl-2′-deoxyuridine (EdU; 50 mg/kg body weight; C00053, RIBOBIO). WAT was harvested at day 7 and fixed in 4% paraformaldehyde (PFA) for further paraffin embedding and section. EdU staining was performed as described by Salic *et al*.^20^ Briefly, 6-μm-thick sections on glass slides were washed twice with 3% bovine serum albumin (BSA) (A8020, Solarbio, China) in PBS for 20 min and permeabilized with 0.5% Triton X-100 (CT11451-100MX, Coolaber, China). After washing, the sections were incubated with a Click-iT™ reaction cocktail including Click-iT™ reaction buffer, 0.5 mM CuSO_4_, 10 μM Alexa Fluor 594-Azide and reaction buffer (C10371-1, RiboBio) for 30 min. Then, the sections were washed once with PBS and incubated with 5 μg/mL Hoechst 33342 for 10 min. All experimental procedures were carried out at room temperature.

### Hematoxylin-eosin and Oil Red O staining

Briefly, tissues samples were fixed in 4% PFA and processed for paraffin embedding. Multiple 6-μm-thick sections of tissues were prepared and stained with hematoxylin and eosin (H&E) for morphological analysis. For Oil Red O staining, frozen liver and muscle tissues sectioned at 8-μm were stained with Oil Red O (O1391-500 ML, Sigma) for 20 min.

## Immunohistochemistry

All tissue specimens were fixed in 4% PFA for 24 h, dehydrated, embedded into paraffin and then sectioned using standard protocols. Multiple 6-μm sections were permeabilized with 0.5% Triton X-100 for 10 min, blocked with 3% BSA for 30 min at room temperature, and then incubated with anti-UCP1 antibody (1:200, ab10983, Abcam) and anti-mitochondrial antibody (1:500, ab28172, Abcam) at 4°C. After 16 h, above sections were washed twice with PBS containing 0.05% Triton X-100 and incubated with secondary antibodies.

### Lipid analysis

Briefly, 0.33 g of liver, muscle, or adipose tissues were homogenized in 0.9 ml of absolute ethanol. After mixing the liquors, the organic phase was collected and centrifuged at room temperature for 30 min at 2000 *g*. The lower organic phases were transferred to clean test tubes and air-dried in a chemical hood overnight. TG concentrations were then measured using a triglyceride reagent (A110-1, Nanjing Jiancheng Bioengineering Institute) according to the manufacturer’s instructions.

### Fecal microbiota analysis

Bacterial genomic DNA from fresh stool samples was immediately extracted using a fecal DNA isolation kit (MP Biomedicals, USA) according to the manufacturer’s protocols and stored at −80°C. PCR amplification of the V3–V4 region of bacterial 16S rRNA genes was then performed using the forward primer 338 F (5′-ACTCCTACGGGAGGCAGCA-3′) and the reverse primer 806 R (5′-GGACTACHVGGGTWTCTAAT-3′). PCR amplicons were purified using Agencourt AMPure beads (Beckman Coulter, Indianapolis, IN) and quantified using the PicoGreen dsDNA Assay Kit (Invitrogen). Subsequently, high-throughput pyrosequencing of the PCR products was performed on an Illumina MiSeq platform at Shanghai Personal Biotechnology Co., Ltd (Shanghai, China).

The raw paired-end reads from the original DNA fragments were merged using FLASH2 program and assigned to each sample based on their unique barcodes. All high-quality reads from one sample were clustered into operational taxonomic units (OTUs) based on a 97% sequence similarity according to UCLUST. Sequence data analyses were performed using QIIME and R packages (v3.2.0). For alpha diversity analysis, we rarified the OTU into several metrics, including curves of OTU rank, rarefaction, and Shannon, and the indexes of Shannon, Chao1, Simpson, and ACE were calculated. For analyzing beta diversity, a heatmap of RDA-identified key OTUs, principal coordinate analysis, non-metric multidimensional scaling, and an unweighted pair group method with arithmetic mean (UPGMA) were performed using QIIME2. The linear discriminant analysis effect size (LEfSe) for quantitative analysis of biomarkers was determined in each group. The abundance of each OTU was normalized by log10 transformation and used to construct redundancy analysis (RDA) models. Statistical significance was determined using the Monte Carlo permutation procedure with 499 random permutations.

### miRNA discovery and profiling

Small RNA-seq was performed to identify microRNAs (miRNAs) difference in WAT between HFD and HFD+B group (n = 3 per group). Briefly, 1 μg of total RNA extracted by Trizol LS (Invitrogen) was used to construct small RNA libraries using the Next Multiplex Small RNA Library Prep Set for Illumina in accordance with the manufacturer’s guidelines. The library was then sequenced on a HiSeq platform (Illumina) by Shanghai Personal Biotechnology Co., Ltd. Data processing was performed by DESeq version 1.18.0 to analyze the differential expression of miRNAs. Differentially expressed miRNAs were screened based on multiple differences (|logFC| ≥2) and significant differences in expression (*P* < .05). Target genes for differentially expressed miRNAs were predicted using two bioinformatics algorithms (TargetScan and miRanda). The data predicted by both algorithms were combined, and overlaps were calculated. Kyoto Encyclopedia of Genes and Genomes (KEGG) pathway enrichment analysis of target genes of differentially expressed miRNAs was performed. In all tests, the *P*-values were calculated using Benjamini-corrected modified Fisher’s exact test. *P*-values <0.05 were considered to be significant.

### Transcriptome sequencing and analysis

Total RNA was extracted using TRIzol reagent (Invitrogen) following the manufacturer’s instructions (n = 3 per group). All complementary DNA (cDNA) libraries were prepared by TruSeq Stranded mRNA LT Sample Prep Kit (Illumina, San Diego, USA). Paired-end reads of 150-bp length were generated after the constructed libraries were sequenced using an Illumina HiSeq X Ten platform. Subsequently, raw reads in FASTQ format were processed using Trimmomatic. Significantly differently expressed genes (DEGs) between two groups were identified using edgeR software version 3.2. The threshold used to screen upregulated or downregulated genes was |logFC| ≥2.0 with a *P*-value <0.05. All processes were performed by Shanghai OE Biotech Co., Ltd (Shanghai, China)

### Gene expression analysis

Total RNA from tissues and cells was extracted using the TRIzol reagent (Invitrogen) in accordance with the manufacturer’s protocols. The quality and concentration of the obtained total RNA were estimated using denaturing gel electrophoresis and a spectrophotometer (Thermo, Waltham, MA, USA). Reverse transcription of mRNAs and miRNAs was performed using a commercial kit (RR036A & 638313, TaKaRa, China), following the manufacturer’s instructions. Quantitative reverse transcription PCR (qRT-PCR) of mRNAs and miRNAs was performed using an SYBR Premix Ex Taq kit (TaKaRa) on a Bio-Rad IQ™5 system (Bio-Rad, Hercules, CA, USA). The relative expression levels of mRNAs and miRNAs were calculated using the 2^−ΔΔCt^ method. The expression levels of individual mRNAs or miRNAs were normalized using *β-actin* or *U6* respectively. Primer sequences used for qRT-PCR are listed in Supplementary Table 1.

### Luciferase reporter assay and promoter methylation analysis

Wild-type (*YY1*-WT) and mutant-type (*YY1-*MUT) 3ʹ-UTRs of *YY1* were inserted at the 3ʹ-end of the coding sequence of Renilla luciferase of the psiCHECK-2 luciferase miRNA expression reporter vector. All plasmids were manufactured by TsingKe Biotech. For luciferase reporter analysis, miR-378a-3p mimics, miR-378a-5p mimics, or the negative control were co-transfected with *YY1*-WT or *YY1*-MUT into HeLa cells, respectively, using Lipofectamine3000 (Invitrogen). After transfection for 24 h, dual-luciferase assay was performed using the Dual-Glo Luciferase Assay System (Promega, Madison, WI, USA) following the manufacturer’s instructions. Renilla luciferase activity was normalized to firefly luciferase activity. To measure the promoter methylation levels of miR-378a, genomic DNA was extracted using the TIANamp Genomic DNA Kit (TIANGEN, China) and DNA methylation at the indicated loci was measured using a mass spectrometry-based method at Beijing Liuhe Huada Gene Science Co.

### Statistical analyses

All quantitative results were presented as the means ± standard error of the mean, and statistical analyses were performed using SPSS software version 21 (Chicago, IL, USA). Differences between two groups were assessed using the unpaired two-tailed Student’s t-test. One-way analysis of variance was performed to compare more than two parametric groups. A *P*-value of <0.05 was considered significant.

## Results

### Gut microbiota orchestrate the anti-obesity function of betaine in mice

**Figure 1.**
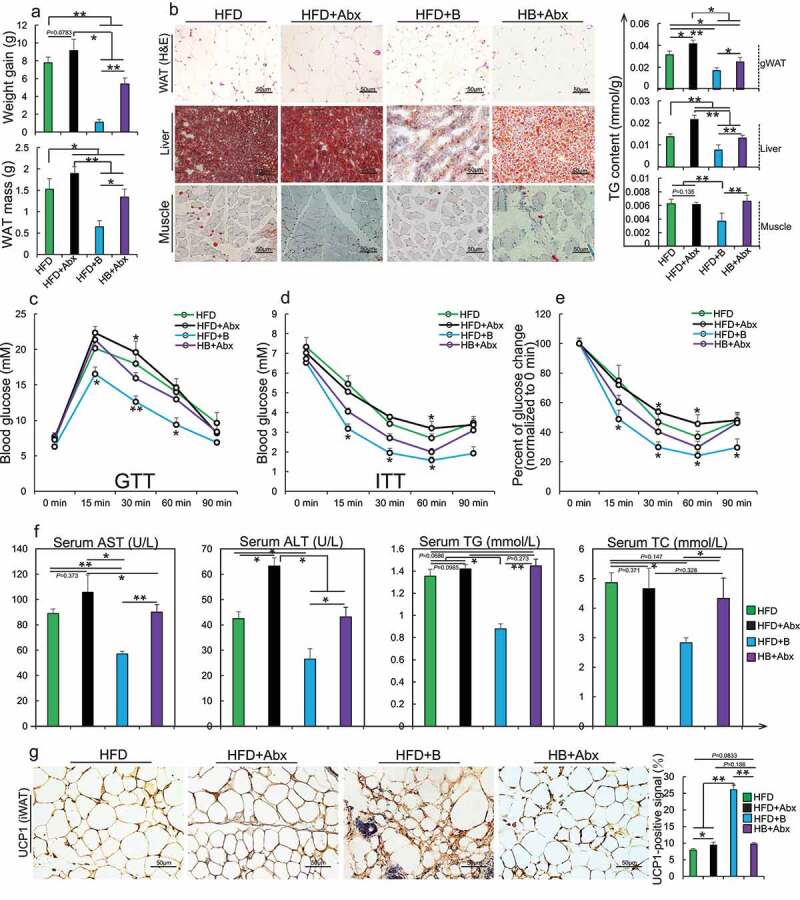
Gut microbiota are involved in obesity prevention by betaine

**Figure 2.**
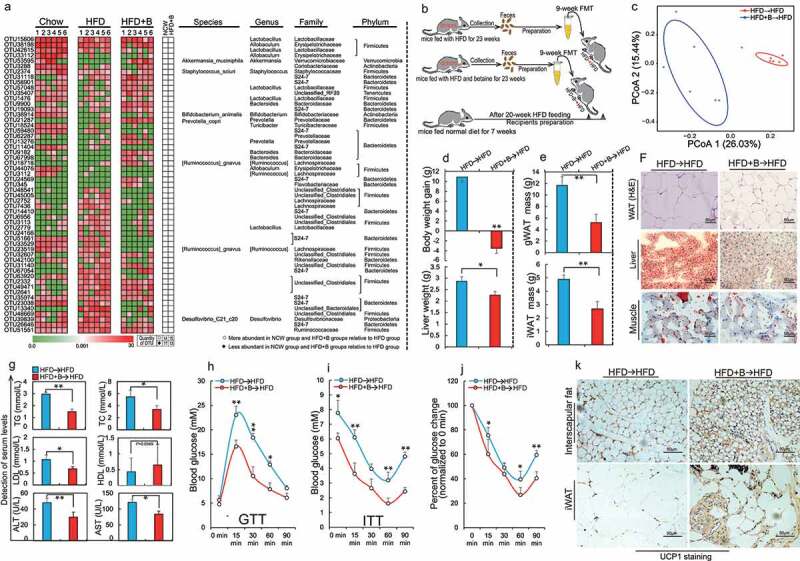
Betaine influences gut microbiota to orchestrate the improvement of obesity and metabolic disorders in mice

Preliminary studies from our and other groups demonstrated that betaine supplementation could reduce HFD-induced obesity.^16,21^ However, its underlying regulatory mechanism remains largely unclear. Given the interrelationships between gut microbes and obesity, and identification of edible extracts from daily foods as contributors to microbiota homeostasis in obese individuals,^14,22^ we investigated whether obesity prevention by betaine is associated with gut microbiota. Interestingly, upon depletion of intestinal microbiota using antibiotics,^17^ betaine not only lost most of its function to prevent HFD-caused development of obesity including increased weight and fat mass (Figure 1a, b), glucose intolerance (Figure 1c-E), and dyslipidemia (Figure 1f) but also failed to stimulate browning of inguinal white adipose tissues (iWAT) (Figure 1g), suggesting that gut microbiota are crucial in betaine-mediated improvement in obesity. To characterize a causal relationship between gut microbiota and betaine, microbiota communities were profiled by 16S rRNA gene sequencing. Beta-diversity analysis showed a distinct clustering of microbiota in each group, with both HFD and betaine significantly altering gut microbiota composition in mice, respectively (Supplementary Figure S1A, B). To further reveal the impact of betaine on gut microbiota, redundancy analysis (RDA) was performed to identify gut microbiota phylotypes responding to betaine. When compared with NCW-fed mice, HFD feeding dramatically altered 215 OTUs, producing 132 decreased OTUs and 83 increased OTUs. Interestingly, we identified 283 different OTUs between NCW-fed mice and NCW+B-fed mice, among which 115 OTUs increased and 168 OTUs decreased in NCW-fed mice after supplementation of betaine. In HFD-fed mice, 80 OTUs were changed in response to betaine treatment (38 increased and 42 decreased) (Supplementary Figure 1C-E, Supplementary Table 2). Notably, some of 80 OTUs have been shown to produce beneficial effects on obesity and obesity-associated complication including *Prevotella*,^23^ *Ruminococcus*,^13^ *Oscillospira*,^24^ *Bifidobacterium*,^25–27^ *Akkermansia muciniphila*,^18,28^ *Lactobacillus*,^29,30^ and *Dorea*.^31,32^ In contrast, *Mucispirillum schaedleri* and *Desulfovibrio* are positively correlate with development of diseases^33–35^ (Figure 2a, Supplementary Table 2). These results implied that prevention of obesity by betaine might be associated with modulation of gut microbiota.

Mice treated with an antibiotic cocktail were fed with HFD or HFD+betaine for 45 days (HFD+Abx, HB+Abx, respectively), after which the following analyses were performed: (a) Body weight gain and white adipose tissues (WAT) (n = 6 per group) were analyzed; (b) Representative H&E staining of gonads white adipose tissue (gWAT) sections, Oil Red O staining of liver and muscle sections (n =  4–5 per section per group), and total triglyceride (TG) content in three tissues were detected (n = 5–6 per group); (c) Glucose tolerance test (GTT, n = 4 per group); (d-e) insulin tolerance test (ITT; n = 4– 5 per group) and rate of change in plasma glucose concentration versus time curve after performing ITT (n = 4– 5 per group) were performed; (f) The serum of aspartate aminotransferase (AST, U/L), alanine aminotransferase (ALT, U/L), TG (mmol/L) and total cholesterol (TC, mmol/L) (n = 4–6 per group) were tested; (g) Representative UCP1 immunohistochemical staining in an inguinal white adipose tissue (iWAT) section (n = 4 per section per group); UCP1-positive signals were analyzed using Image J software. Data are shown as means ± SEM. Statistical analyses were performed using one-way ANOVA test; *, *P* < .05; **, *P* < .01.

Seven-week old mice were fed with (1) Chow, (2) HFD or (3) HFD+betaine (HFD+B) for 23 weeks. (a) Heat maps showing the abundance of bacterial taxa significantly altered by betaine in HFD-fed mice. Bacterial taxa (phylum, class, order, family, genus, and species) are shown in the right panel. White circles and black diamonds indicate bacterial taxa that are increased and decreased in the normal chow-fed and HFD+B-fed groups, respectively, when compared with the HFD-fed group (n = 6 per group); (b) A specimen of fecal transplant: 7-week-old mice were fed with HFD for 20 weeks and then selected as recipients; mice fed with HFD or HFD+betaine for 23 weeks were selected as donors of fresh feces samples. Subsequently, fecal samples collected daily from these two donors were prepared as described in methods and transplanted daily (100 μl per mice) by oral gavage into recipients for 9 weeks (n = 8–10 per group). These recipients are defined as HFD→HFD group and HFD+B→HFD group, respectively. After FMT for 9 weeks the following parameters were analyzed: (c) PCoA plots showing two diet groups defined by unweighted UniFrac microbiota analysis (n = 6 per group); (d) Weight gain and liver weight (n = 6 per group); (e) Mass of gonadal white adipose tissues (gWAT) and inguinal white adipose tissues (iWAT) (n = 5–6 per group); (f) Representative hematoxylin-eosin staining of gWAT sections and Oil Red O staining of liver and muscle sections (n = 4–5 per section per group); (g) The serum levels of total triglycerides (TG, mmol/L) and total cholesterol (TC, mmol/L), high-density lipoprotein cholesterol (HDL, mmol/L), low-density lipoprotein cholesterol (LDL, mmol/L), aspartate aminotransferase (AST, U/L) and alanine aminotransferase (ALT, U/L) (n = 4–5 per group); (h) Glucose tolerance test (GTT, n = 6 per group); (i) Insulin tolerance test (ITT; n = 5–6 per group) and (j) the rate of change in plasma glucose concentration versus time curve after performing ITT (n = 6 per group); (K) Representative UCP1 immunohistochemical staining of interscapular fat and iWAT section (n = 4 per section per group). Data are shown as means ± SEM. Statistical analyses were performed using LEfSe test (A) and Student’s *t*-test (D, E, and G-J); *P < .05; **P < .01.

It is known that the ability of the gut microbiota to mediate obesity can be transferred to other animals.^12,36^ In order to test whether betaine-altered gut microbiota can alleviate obesity, and FMT were performed (Figure 2b). It is not surprising that a marked difference in microbiota composition was observed between two recipient groups after a 9-week FMT (Figure 2c, Supplementary Figure 2A-C and Supplementary Table 3). Indeed, multiple betaine fecal transplants-altered OTUs such as *Bacteroides,^37^ Prevotella,^23^^,^^38^ Parabacteroides,^11^* and *Corynebacterium^39^* have been reported to be mainly involved in pathways associated with regulation of energy and glucose metabolism (Supplementary Figure 2D). In further support of our hypothesis, FMT from HFD+B-fed mice resulted in reduction in WAT mass (Figure 2d-f), lipid accumulation in non-adipose tissues (Figure 2f), dyslipidemia (Figure 2g) and glucose intolerance (Figure 2h-j). In agreement with the results of betaine supplementation, FMT from HFD+B-fed mice also caused a slight increase in circadian core body temperature (Supplementary Figure 3A, B) along with increased activation of BAT and browning of iWAT (Figure 2k, Supplementary Figure 3C-F). To adequately understand underlying mechanism by which betaine-altered gut microbiota reduced obesity, high-throughput sequencing was further performed. Approximately 717 differentially expressed genes (log2FC ≥ 2-fold, *P*< .05; 289 upregulated and 428 down-regulated) in liver were found between two recipient groups (Supplementary Figure 3g, Supplementary Table 4). Consistent with the altered metabolic phenotype, these differentially expressed genes were primarily related to regulation of energy homeostasis and glucose metabolism (Supplementary Figure 3H), such as *Park7* (log2FC = 15.7, *P*= 8.43E-08), *Flot1* (log2FC = 13.3, *P*= 1.61E-06), *Gdpd3* (log2FC = −6.6, *P*= 3.33E-06), *CTSK* (log2FC = −9.3, *P*= .0008) and *CTLA-4* (log2FC = −6.78, *P*= .007). Taken together, these results suggest that gut microbiota are required for betaine-mediated prevention of obesity and obesity-associated metabolic comorbidities.

### Betaine improves obesity and MS through gut microbiota-producing SCFAs

**Figure 3.**
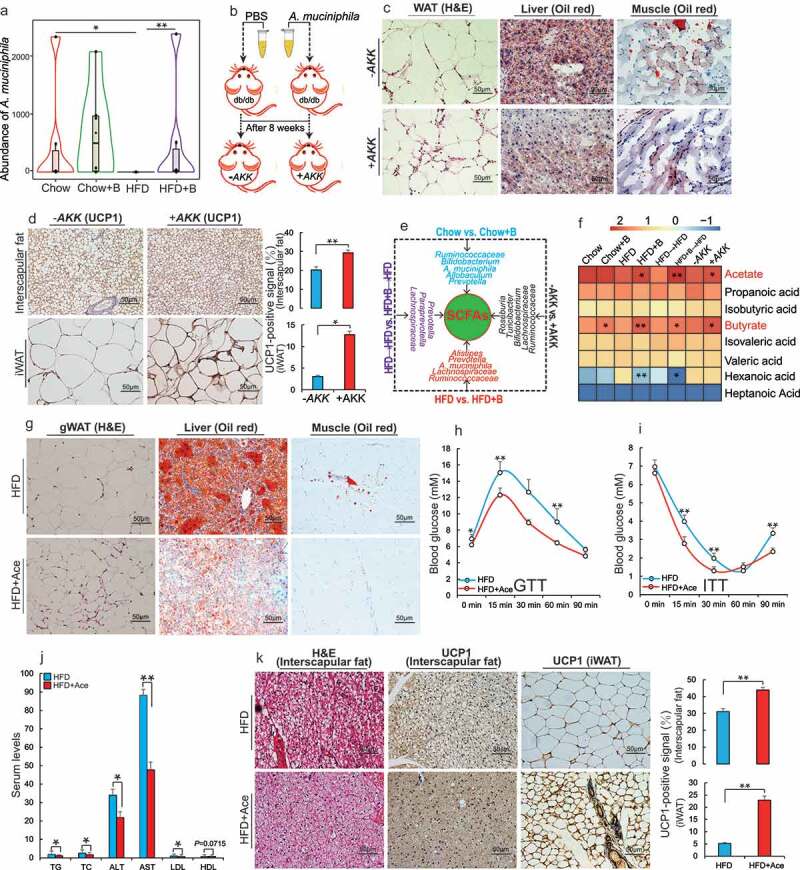
Betaine improves obesity and related metabolic disorders through SCFAs produced by gut microbiota

After having found a strong causal relationship between gut microbiota and betaine during obesity treatment, we aimed to identify how betaine-altered gut microbiota reduce obesity and obesity-associated metabolic comorbidities. The mucus layer is a protective barrier and contributes to support beneficial endogenous microbiota adapted for symbiotic living as a niche in the intestine, while previous studies showed a significant impairment of mucus layer along with the intake of HFD.^40,41^ Among bacteria affected by betaine, *A. muciniphila* was significant as it is a key species of mucus layer and maintains beneficial mucosal microbial networks.^40^ Interestingly, we found an increase in *A. muciniphila* culturing in standard medium containing betaine, and HFD+B mice showed a similar abundance of *A. muciniphila* to that of Chow-fed mice (Figure 3a, Supplementary Figure 4A, B), which may reveal its potential functions as a contributor of *A. muciniphila* growth. Previous studies have exhibited a marked decrease in *A. muciniphila* in individuals with obesity and T2DM, and its increase can reduce the development of obesity and obesity-associated complication.^42,43^ To verify the role of *A. muciniphila* in the prevention of obesity by betaine, we first investigated whether supplementation with *A. muciniphila* exhibits similar effects on obese mice. Consistent with negative correlation between *A. muciniphila* and obesity,^18,28,44^ Chow-fed mice supplemented with *A. muciniphila* showed lower weight gain (Supplementary Figure 4C) and higher anti-obesity capacity under HFD conditions (Supplementary Figure 4D-M). Lepr^db/db^ is a classical mouse model for obesity and associated MS. Similar to both betaine and betaine-FMT administration, supplementation with *A. muciniphila* also reduced obese and metabolic phenotypes (Figure 3b, Supplementary Figure 5A-F) of Lepr^db/db^ mice. Increasing BAT mass and UCP1 signals were also found in both the interscapular region and iWAT (Figure 3d), which was consistent with prevention of *A. muciniphila* on HFD-induced obesity.^42^

(a) The abundance of *Akkermansia muciniphila* at the genus level in the gut after normal chow (Chow) or high-fat diet (HFD) fed mice were supplemented with betaine in water for 23 weeks (Chow+B and HFD+B, respectively) (n = 6 per group). (b) After *A. muciniphila* or PBS was administered to Lepr^db/db^ mice by oral gavage for 8 weeks (+AKK and -AKK, respectively), (c) Representative hematoxylin-eosin (H&E) staining of gonadal white adipose tissues (gWAT) sections and Oil Red O staining of liver and muscle sections (n = 4 per section per group); (d) Representative UCP1 immunohistochemical staining of interscapular fat and inguinal white adipose tissue (iWAT) section (n = 4 per section per group), UCP1-positive signals were analyzed using Image J software; (e) Illustration of differential intestinal bacteria that produces short-chain fatty acids (SCFAs) between eight groups; (f) Heat maps showing the SCFA composition in feces (log10 [FPKM], n = 5–6 per group). HFD-fed mice were supplemented with 150 mM sodium acetate (HFD+Ace) for 45 days and the following analyses were performed: (g) Representative H&E staining of (gWAT) sections or Oil Red O staining of liver and muscle (n = 4 per section per group); (h) Glucose tolerance test (GTT, n = 6 per group); (i) Insulin tolerance test (ITT, n = 5 per group); (j) Detection of serum levels of total serum triglycerides (TG, mmol/L), total cholesterol (TC, mmol/L), alanine aminotransferase (ALT, U/L), aspartate aminotransferase (AST, U/L), low-density-lipoprotein cholesterol (LDL, mmol/L), and high-density-lipoprotein cholesterol (HDL, mmol/L) (n =  4– 6 per group); (k) Representative H&E staining of interscapular fat sections and UCP1 immunohistochemical staining of interscapular fat and iWAT section (n = 4 per section per group); UCP1-positive signals were analyzed using Image J software. Data are shown as means ± SEM. Statistical analyses were performed using Student’s *t*- test; *P < .05; **P < .01.

(Figure 4b).

**Figure 4.**
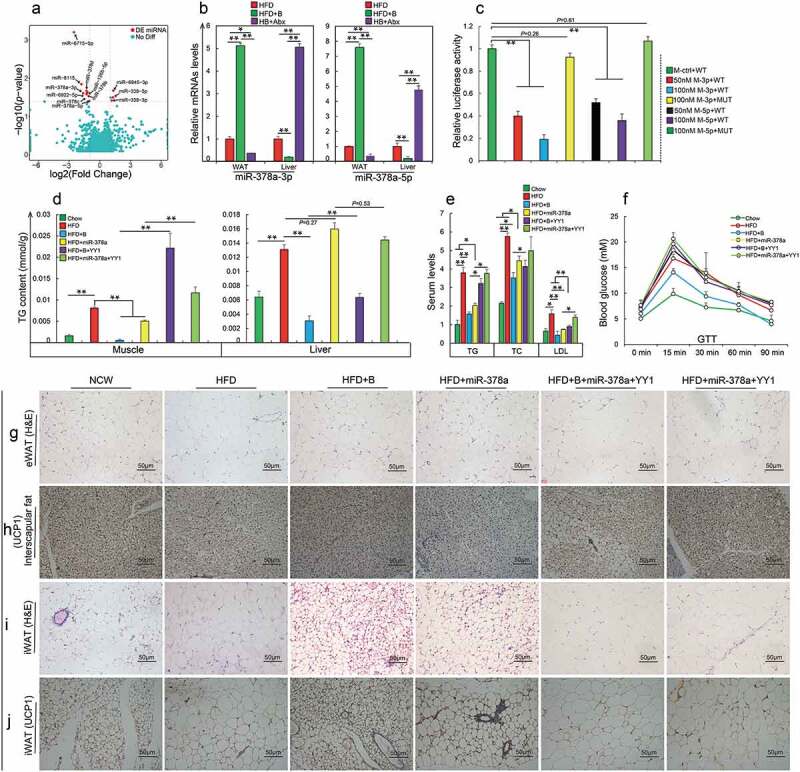
Betaine prevents obesity by regulating gut microbiota-derived miR-378a family

(a) Volcano plot of differentially expressed miRNAs in inguinal white adipose tissue (iWAT) in mice fed with high fat diet (HFD), or HFD and betaine (HFD+B); (b) qRT-PCR analysis of miR-378a family mRNA levels in iWAT and liver after gut microbiotas in HFD+B-fed mice were eliminated by an antibiotic cocktail (HB+Abx, n = 3–4 per group); (c) Luciferase assay revealed the suppressive effect of miR-378a-3p/5p on the activity of 3ʹ-UTR of YY1 (M-ctrl: mimics negative control; M-3p: miR-378a-3p mimics; M-5p: miR-378a-5p mimics; WT: wild-type 3ʹ-UTR of *YY1*; MUT: mutant-type 3ʹ-UTR of *YY1*; n = 6 per group); After 1) HFD-fed mice or HFD+B-fed mice were injected with miR-378a-3p/5p precursor or *YY1* (HFD+miR-378a and HFD+B+ YY1, respectively); 2) HFD+miR-378a were injected with *YY1* plasmids (HFD+miR-378a+YY1), and the following analyses were performed: (d) Detection of triglycerides (TG) levels of muscle and liver (n = 3– 5 per group); (e) Detection of serum TG, cholesterol (TC) and low-density-lipoprotein cholesterol (LDL) (n = 3–4 per group); (f) Glucose tolerance test (GTT, n = 4–5 per group); (g) Representative hematoxylin-eosin (H&E) staining in gonadal white adipose tissue (gWAT) sections (n = 3 per section per group); (h) Representative UCP1 immunohistochemical staining in interscapular fat section (n = 3 per section per group); (i) Representative H&E staining in iWAT sections (n = 3 per section per group); (j) Representative UCP1 immunohistochemical staining in iWAT sections (n = 3 per section per group). Data are shown as means ± SEM. Statistical analyses were performed using one-way ANOVA test (B-E);*P < .05; **P < .01.

In agreement with a crucial role of *A. muciniphila* in maintaining microbiome composition,^40^ we also observed a clear alteration of gut microbiome structure in Lepr^db/db^ mice after supplementation with *A. muciniphila* (Supplementary Figure 6A). Interestingly, four of these microflora increased by supplementation with *A. muciniphila* have been identified as producers of SCFAs^45^ (Figure 3e, Supplementary Figure 6B). This observation supported previous reports that the regulation of host metabolism by *A. muciniphila* is associated with the production of metabolites such as SCFAs.^40^ Similarly, abundant SCFA-producing bacterial genera were also identified in feces of mice receiving betaine or betaine-FMT (Figure 3e, Supplementary Table 2, 3). It is well recognized that SCFAs are the most abundant gut microbial metabolites and are crucial for regulating host energy metabolism.^45^ These results led us to hypothesize that betaine prevents the development of obesity by modulating the production of SCFAs. To test this hypothesis, we examined the SCFA concentrations in fecal samples from different mouse models. When compared with the corresponding control groups, almost all treatments caused an increase in SCFA concentration (Supplementary Figure 6C). Consistently, elevated expression of two key receptor molecules GPR43/41 for microbial SCFAs as well as their well-known downstream targets (*ERK2, P38* and *JNK1/2*)^46,47^ showed in mice after supplementation with betaine or betaine-altered gut microbiota (Supplementary Figure 6D). Given that overexpression of *GPR43* in adipose tissues can protect against HFD-induced obesity and MS,^48^ these results suggest that protection against to obesity by betaine may be linked to microbial SCFAs.

SCFAs are a subgroup of fatty acids. In order to adequately characterize the role of betaine in regulating the production of microbial SCFAs, we identified specific SCFAs responding to betaine-related treatment by analyzing SCFA composition. Interestingly, although propionate, acetate and butyrate were the most abundant microbial SCFAs in the feces^45^ (Supplementary Figure 6E), only acetate and butyrate showed a significant increase after treatment with betaine, betaine-FMT or *A. muciniphila* (Figure 3f). This observation was consistent with significantly increased abundance of four critical butyrate or acetate producers: *A. muciniphila, Ruminococcus, Oscillospira*, and *Lactobacillus* (Supplementary Figure 6B, Supplementary Table 2, 3). Previously, the potential effect of acetate and butyrate on obesity was only partially investigated. In agreement with our results obtained after betaine supplementation and betaine-FMT, studies conducted in mice and humans showed a marked improvement of gut microbial butyrate on obesity and insulin resistance.^49–51^ This response may be associated with BAT activation, enhanced mitochondrial function, reduced energy absorption in the small intestine, and increased energy expenditure.^49,50^ However, it is worth noting that the function of acetate on obesity and glucose homeostasis is controversial. To gain further insight into the impact of acetate on obesity and metabolism, we fed mice under HFD conditions with acetate in water. Similar to the results in mice fed with HFD and butyrate (HFD+But; Supplementary Figure 7), supplementation with acetate significantly reduced weight gain (Supplementary Figure 8A) and improved lipid metabolism (Figure 3g, Supplementary Figure 8B-E), glucose homeostasis (Figure 3h, I) and dyslipidemia (Figure 3j) of HFD-fed mice, which was consistent with a previous report.^52^ In particular, supplementation with acetate clearly inhibited HFD-induced BAT hypertrophy, and enhanced the processes of energy dissipation, as evidenced by increased activation of BAT (Figure 3k) and mitochondrial signals (Supplementary Figure 8 F) in mice fed with HFD and acetate. This effect was similar to these observations in HFD-fed mice after treatment with betaine, betaine-FMT, and *A. muciniphila*. Therefore, together with the findings that colonic acetate infusions can promote fat oxidation and improve metabolic markers of individuals with obese,^53,54^ these results reveal that gut microbial SCFAs such as butyrate and acetate are important for improvement of obesity and obesity-associated MS by betaine.

### Gut microbial butyrate and acetate prevent obesity and MS by regulating host miR-378a family

Ejaz *et al*.^16^ previously reported that dietary betaine increases Fgf21 levels to improve metabolic health of mice. To identify potential downstream factors of betaine-altered gut microbiota in reducing obesity, we measured Fgf21 in both WAT and hepatic tissues, the two main producers of Fgf21. Consistent with a previous finding that gut microbiota did not influence Fgf21,^55^ no significant difference in HFD-fed mice was observed after gut microbiota deletion (Supplementary Figure 9), implying that Fgf21 was not involved in the regulatory mechanism by which betaine prevented obesity by influencing gut microbes. Recently, gut microbial SCFAs were identified as modulators of epigenetic modifications for host metabolism.^47^ As important constituents of epigenetics, miRNAs also have critical roles in various physiological homeostasis mechanisms, including glucose and lipid metabolism.^56^ Given that more than 95% of microbial SCFAs are absorbed by the host, we hypothesized that betaine-mediated microbial butyrate and acetate alleviated obesity is associated with regulation of host miRNAs. As shown in Figure 4a, we identified 12 differentially expressed miRNAs in iWAT upon comparing HFD-fed mice with HFD+B-fed mice (|log2FC| ≥2, *P*< .05). Although these miRNAs were strongly involved in energy or glucose metabolism pathways (Supplementary Figure 10A), only three miRNAs including miR-6715-5p, miR-378a-3p, and miR-378a-5p showed a marked response to these betaine-associated treatments (Supplementary Figure 10B). Considering the extremely low abundance of miR-6715-5p, and the fact that both miR-378a-3p and miR-378a-5p are encoded by the first intron of PGC-1β, which is a transcriptional co-activator for controlling energy homeostasis and glucose metabolism,^57^ we focused exclusively on the miR-378a family. As expected, depletion of gut microbiota significantly reversed HFD-altered expression levels of miR-378a-3p and miR-378a-5p, indicating that the miR-378a family might be regulated by gut microbiota. Furthermore, consistent with previous reports,^58,59^ the miR-378a family not only contributed to reduction of HFD-induced adipogenesis (Supplementary Figure 11A, B) but also induced activation of BAT and browning of iWAT in HFD-fed mice (Supplementary Figure 11 C-E). However, in contrast to betaine-improved insulin resistance, miR-378a did not reduce glucose intolerance under HFD conditions (Supplementary Figure 11 F, G), and promoted liver steatosis and hepatic triglyceride in HFD-fed mice (Supplementary Figure 11 H). Taken together, these results suggest that miR-378a family participates in betaine-mediated obesity prevention.

Notably, we observed differential influence of betaine on miR-378a family between WAT and hepatic tissues (Figure 4b). This difference was consistent with previous findings, in which miR-378a was highly expressed in liver but showed lower expression in WAT individuals with obesity or type 2 diabetes.^60,61^ Indeed, increased miR-378a in both hepatic tissue and adipose tissues usually has an opposite effect on obesity.^58,59,61–63^ To better understand the mechanisms by which betaine prevents obesity, we next investigated how betaine regulates miR-378a family through gut microbiota in WAT and liver tissues. It is known that gut microbes-producing acetate and butyrate are important epigenetic regulators of host metabolism, and these miRNAs with hypermethylated CpG islands in their promoters always show lower levels.^64^ In addition to a 536-bp CpG island located at position 848–1383 upstream of the transcription start site of miR-378a (Supplementary Figure 12A), qRT-PCR analysis showed that the expression levels of miR-378a family could be altered by the DNA methylation inhibitor 5-Aza-dC (Supplementary Figure 12B) or the inhibition of DNA methyltransferase (*Dnmt3a, Dnmt3b*, and *Dnmt1*) with siRNAs (Supplementary Figure 12 C, D). We therefore asked whether betaine-mediated regulation of the miR-378a family during obesity prevention is also associated with DNA methylation. In agreement with opposite expression patterns between WAT and liver, HFD-fed mice showed higher levels of promoter methylation of miR-378a in WAT, by contrast, its level was lower in liver compared to that of Chow-fed mice. Almost all treatment including betaine, betaine-FMT, *A. muciniphila*, acetate or butyrate could reduce HFD caused alteration in DNA methylation of miR-378a promoter between these two tissues. Most notably, microbiota disruption also reduced the effect of betaine on promoter methylation of miR-378a in both WAT and liver of HFD-fed mice (Supplementary Figure 12E), revealing that betaine may affect miR-378a family via DNA methylation during prevention of HFD-induced obesity.

miRNAs are known to modulate biological processes through base pairing with the 3′-untranslated regions (3′UTR) of the target mRNAs.^58^ Among these potential target genes of miR-378a family, Yin Yang 1 (*YY1*) was regulated by gut microbiota and showed an expression pattern opposite to that of the miR-378a family (Supplementary Figure 13A-J). To identify whether betaine-derived gut microbiota/miR-378a family improved obesity through *YY1*, we first examined the link between *YY1* and miR-378a family. As expected, the expression of *YY1* could be negatively regulated by the miR-378a family (Supplementary Figure 13 K-M). Overexpression of miR-378a family significantly repressed *YY1* activity by binding to their 3′-UTR (Figure 4c), suggesting that *YY1* was a direct target gene of miR-378a family. Supporting previous findings that *YY1* contributes to HFD-induced obesity,^65–68^ improvement in lipid metabolism (Figure 4d-e) and glucose homeostasis (Figure 4f) by betaine or miR-378a family was obviously reduced by increasing *YY1*. Furthermore, increasing *YY1* almost abolished betaine- or miR-378a family-mediated lipogenesis inhibition (Figure 4g), BAT inactivation (Figure 4h), and browning of iWAT (Figure 4i, j) in HFD-fed mice. These results therefore implicate that *YY1* as an important player for improvement of obesity and MS by betaine-derived gut microbiota/miR-378a family.

## Discussion

Betaine is a natural substance which is wildly present in daily foods including spinach, shellfish, and particularly rich in whole grains.^16^ Previous results indicated that gut microbiota may contribute to production of betaine in mice fed with bran-enriched diets, and reduced plasma levels of betaine in human is positively associated with increased risks of metabolic disorders such as insulin resistance, coronary artery disease, and obesity.^16,69,70^ However, its underlying mechanism is still poorly understood. In the present study, we showed that gut microbiota was crucial for the function of betaine on protecting against development of diet-induced obesity as well as obesity-associated complication, reasoning that supplementation of betaine failed to improve lipid and glucose metabolism, and stimulate browning of WAT of HFD-fed mice.

Previous studies from animals and humans have shown a significant alteration of gut microbiota in obese individuals when compared with normal individuals, deficiency, or dysbiosis in gut microbiota is involved in the development of obesity and MS.^14,22^ Similar to some edible extracts from daily foods,^14,71^ our current findings suggested that supplementation of betaine strongly altered the composition of gut microbiota in HFD-fed mice. Notably, there has been an explosion of interest in the functional role of some bacteria in intestinal tract. Among probiotics, *Prevotella*,^23^ *Ruminococcus*,^13^ *Oscillospira*,^24^ *Bifidobacterium*,^25–27^ *Akkermansia muciniphila*,^18,28^ *Lactobacillus*^29,30^ and *Dorea*^31,32^ have been identified as important contributors of obesity prevention, which by regulating lipid metabolism, glucose uptake and immune response. *Desulfovibrio* is a main producer of hydrogen sulfide and frequently detected in feces of patients with abscesses, which is positively related to mucositis and hyperlipidemic.^34,35,72^ Similarly, one of the abundant inhabitant of the intestinal mucus layer (*Mucispirillum schaedleri*) has also been identified as a pathobiont associated widely with inflammation and oxidative stress.^33^ Supporting our and other findings that betaine can protect against HFD-induced obesity and metabolic disorders,^16,21,73^ we found increased abundance of *Prevotella, Ruminococcus, Oscillospira, Bifidobacterium, Akkermansia muciniphila, Lactobacillus*, and *Dorea*, but a significant decrease in relative abundance of these two pathobionts including *Desulfovibrio* and *Mucispirillum schaedleri* in HFD-fed mice after betaine supplementation. Given that ability of the gut microbiota to mediate obesity can be transferred to other animals,^12,36,71^ we also identified the role of betaine-altered gut microbiota in obese mice by performing FMT. Consistently, the transplantation of feces from HFD+B-fed mice not only significantly modulated gut microbiota composition and transcriptome levels in obese mice, but also obviously improved their adiposity and insulin resistance. Of note, among the top-upregulated genes, *Park7* is noteworthy because it is closely associated with human T2DM, and deficiency of *Park7* results in glucose intolerance and dysregulation of oxidative stress.^74^ *Flot1* was found to be consistently repressed in the liver of individuals with obesity, hyperphagia, and T2DM. Decreased *Flot1* in hepatic tissue may contribute to metabolic dyslipoproteinemia.^75^ In the list of significantly downregulated genes, it was reported that inhibition of *Gdpd3* and *CTSK* showed an anti-obesity effect and significant improvement of liver steatosis and inflammation.^76–78^ In addition, *CTLA-4* influenced by microbial exposure has also been identified as a key target of immunotherapy, and anti-CTLA-4 antibodies are used widely to reduce obesity-associated complications such as cancers, diabetes, and inflammation.^79,80^ Therefore, our results demonstrated directly that by betaine may prevents obesity by modulating the composition of the gut microbiota.

SCFAs are the main metabolites produced by gut microbiota. It was reported that increasing SCFAs in intestinal tract not only enhances host energy expenditure and reverses HFD-induced MS via a PPARγ-dependent switch from lipogenesis to fat oxidation^81,82^ but can also improve inflammation by modulating regulatory T cells via histone deacetylase inhibition and suppressing the secretion of pro-inflammatory cytokines.^83^ In contrast, deficiency of SCFAs is positively associated with the development of T2DM, increased adiposity, and obesity.^36^ In our model, we identified abundant SCFA-producing bacterial genera in intestinal tract of HFD+B-fed mice or obese mice receiving feces from HFD+B-fed mice. In particular, supplementation of betaine almost revised SCFAs altered by HFD feeding, suggesting that protection against to obesity by betaine may be linked to microbial SCFAs. SCFAs are a subgroup of fatty acids. However, we here only found a significant increase in acetate and butyrate after betaine-associated treatment. It is known that acetate and butyrate are two kinds of the most abundant microbial SCFA in the feces, and can be largely absorbed by the host.^45^ Previous studies have highlighted the importance of acetate and butyrate in treatment for obesity and obesity-related metabolic disorders.^45,49,52^ In our study, we not only confirmed these findings but also demonstrated that similar to butyrate, acetate also contributed to inhibit HFD-induced inactivation of BAT and stimulate browning of WAT. MicroRNAs play an important role in development of obesity and obesity-associated disease.^84^ Most recently, a study reported that gut microbiota mediates adipogenesis via miR-181.^85^ Similarly, we also found a marked difference in miRNAs in WAT between HFD-fed mice and HFD+B-fed mice, and SCFAs produced by betaine-altered gut microbiota may affect miR-378a family via regulating DNA methylation of its promoter. In particular, overexpression of *YY1*, a direct target gene of miR-378a family, significantly abolished the impact of betaine-related treatment on lipids metabolism. However, in contrast to betaine-improved insulin resistance, miR-378a did not reduce glucose intolerance under HFD conditions. Previous studies showed that elevated miR-378a in WAT counteracts obesity by enhancing lipolysis and thermogenesis, ^(58, 59)^ whereas elevated miR-378a in hepatic tissue impairs glucose homeostasis.^61–63^ Considering increased liver steatosis and hepatic triglyceride in obese mice by miR-378a, this is most likely because the impairment of hepatic insulin signaling by miR-378a counteracts adipose miR-378a-improved glucose homeostasis. In addition, there are several limitations to our study. It is unclear whether betaine has an obvious therapeutic effect for obesity and obesity-associated complication in human. Based on systematic overexpression of miR-378a and *YY1 in vivo*, whether hepatic and adipose tissues are only targets of microbiota-derived signals during prevention of betaine on obesity remains largely unknown. Future work also needs to establish whether the miR-378a family is targetable in humans.

**Figure 5.**
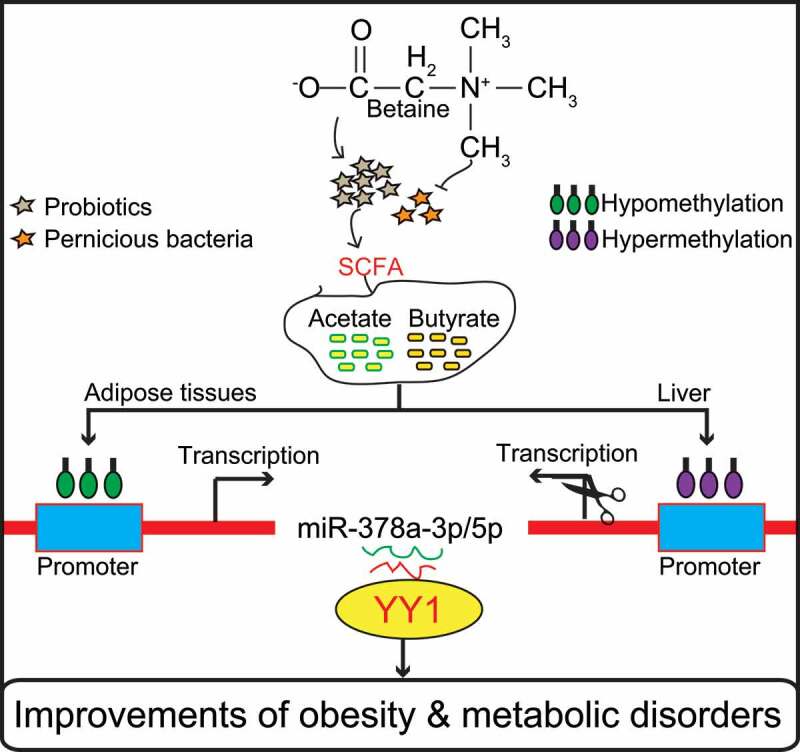
Model of betaine prevents obesity and associated metabolic disorders

In summary, the results of this study show that dietary betaine alleviates gut microbiota imbalance in obese mice and reduces the development of obesity and obesity-related complications, such as NAFLD, inflammation, insulin resistance, and dyslipidemia. Regulation of the miR-378a-*YY1* regulatory axis by gut microbial acetate and butyrate is a critical mechanism for modulating WAT browning, classical BAT activation, and lipid and glucose homeostasis in obese mice after betaine supplementation (Figure 5). These findings offer novel insights into the underlying mechanisms by which gut microbiota affect host metabolism and host immune system, and demonstrate that the betaine-gut microbiota-derived signal axis is a potential therapeutic target in obesity and MS.

## Supplementary Material

Supplemental Material
