# Supplementary material for: Dietary betaine prevents obesity through gut microbiota-drived microRNA-378a family
Source: Gut Microbes. 2021 Feb 8;13(1):1862612. doi: 10.1080/19490976.2020.1862612 (PMC7889173; doi:10.1080/19490976.2020.1862612)
Supplement: Supplemental Material [file KGMI_A_1862612_SM6571.zip › supplementary/Legend-supplementary Figures.docx]

**Sup Figure 1. Betaine supplementation alters gut microbiota of mice.**

Mice fed normal chow (Chow) or a high-fat diet (HFD) were treated with betaine for 23 weeks (Chow+B and HFD+B, respectively), and fecal samples were collected from each diet group for pyrosequencing analysis of bacterial 16S rRNA. (A) PCoA plots showing four diet groups defined by unweighted UniFrac microbiota analysis; (B) Non-metric multidimensional scaling (NMDS) score plot based on Bray-Curtis. (C-E) Redundancy analysis (RDA) comparing OTUs between the Chow and HFD-fed groups (D); Chow vs. Chow+B-fed mice (E); HFD vs. HFD+B-fed mice (F).

**Sup Figure 2. Fecal transplantation alters gut microbiota in mice.**

Fecal material from HFD-fed mice or HFD+B-fed mice was transferred to HFD-induced obese mice (DIO) for 9 weeks (HFD→HFD group and HFD+B→HFD group, respectively), and gut microbiota composition was determined by pyrosequencing analysis of bacterial 16S rRNA. (A) Non-metric multidimensional scaling (NMDS) score plot based on Bray-Curtis; (B) Unweighted UniFrac cluster tree based on the unweighted pair-group method with arithmetic means (UPGMA); (C) Redundancy analysis (RDA) comparing OTUs between two groups; (D) Heat map showing the abundance of bacterial taxa significantly altered by fecal transplantation. Bacterial taxa (phylum, class, order, family, genus, and species) are shown in the right panel. White circles and black diamonds indicate bacterial taxa with increased and decreased numbers in DIO mice receiving fecal transplantation from HFD or HFD+B-fed mice, respectively.

**Sup Figure 3. Fecal transplantation alters genes involved in lipid and glucose metabolism.**

Fecal materials from mice fed with a high fat diet (HFD) or with HFD and betaine (HFD+B) were transferred to diet-induced obese (DIO) mice for 9 weeks (HFD→HFD group and HFD+B→HFD group, respectively), (A) Circadian rectal temperature on the day of feeding (n=4-5 per group); (B) qRT-PCR analysis of mRNA levels of Dio2 and PGC1α in muscle, liver and inguinal white adipose tissue (iWAT) (n=4 per group); (C) Interscapular fat mass (n=6 per group); (D) qRT-PCR analysis of mRNA levels of UCP1 in interscapular fat (n=3 per group); (E, F) qRT-PCR analysis of mRNA levels of PRDM16, TBX1, UCP1, UCP3 and Cidea in iWAT (n=3 per group); (G) Volcano plot of differentially expressed genes (DEGs) in liver, and qRT-PCR analysis of mRNA levels of Park7, Flot1, CTSK, Gdpg3 and CTLA-4 (n=3 per group); (H) KEGG pathway enrichment analysis of DEGs. Data are shown as means ± SEM. Statistical analyses were performed using Student’s t test (A-F) and quasi-likelihood F-tests in R package edgeR (G); **P* < 0.05; ***P* < 0.01.

**Sup Figure 4. *Akkermansia muciniphila* supplementation improves obesity and related metabolic disorders in mice.**

(A) Abundance of *Akkermansia muciniphila* at the genus levels in gut after mice fed normal chow (Chow) or high fat diet (HFD) were supplemented with betaine in water for 23 weeks (Chow+B and HFD+B, respectively; n=6 per group); (B) *A. muciniphila* concentrations after 24 h-culture in standard medium containing different concentrations of betaine were measured in a spectrophotometer 600 nm wavelength (n=6-8 per group). (C) Body weight gain after mice under normal chow (Chow) were supplemented with *A. muciniphila* (Chow+AKK) for 8 weeks (n=5-6 per group). Chow-fed mice and Chow+AKK mice were fed with HFD for 5 weeks (Chow→HFD, Chow+AKK→HFD; n=5-6 per group), and the following analyses were performed: (D) Weight gain; (E) Mass of inguinal white adipose tissue (iWAT); (F) Mass of gonads white adipose tissue (gWAT); (G, H) Serum levels of total triglycerides (TG, mmol/L; n=4 per group) and total cholesterol (TC, mmol/L; n=4-5 per group); (I) Liver weight (n=5 per group); (J) Representative H&E-stained paraffin-embedded sections of liver (n=3 per section per group); (K) Serum levels of aspartate aminotransferase (AST, U/L; n=4 per group), alanine aminotransferase (ALT, U/L; n=4 per group); (L) Glucose tolerance test (GTT, n=5 per group); (M) Representative UCP1 immunohistochemical-stained paraffin-embedded sections of interscapular fat and iWAT (n=3 per section per group). Data are shown as means ± SEM. Statistical analyses were performed using Student’s t test; **P* < 0.05; ***P* < 0.01.

**Sup Figure 5. The effect of supplementation with *Akkermansia muciniphila* on** Lepr^db/db^ mice.

Lep^db/db^ mice were supplemented with *A. muciniphila* or not for 8 weeks (+*AKK* and -*AKK*, respectively), and the following analyses were performed: (A) Body weight gain and liver weight (n=4-5 per group); (B) Triglycerides (TG) content in three metabolic tissues (n=4 per group); (C) Serum levels of TG (mmol/L) and total cholesterol (TC, mmol/L), high-density lipoprotein cholesterol (HDL, mmol/L), low-density lipoprotein cholesterol (LDL, mmol/L), aspartate aminotransferase (AST, U/L) and alanine aminotransferase (ALT, U/L) (n=3-4 per group); (D) Glucose tolerance test (GTT, n=3-4 per group); (E) Insulin tolerance test (ITT, n=4 per group); (F) Percent change in blood glucose concentration versus time curve after ITT (n=4 per group). Data are shown as means ± SEM. Statistical analyses were performed using Student’s t test; **P* < 0.05; ***P* < 0.01.

**Sup Figure 6. Betaine supplementation regulates the production of SCFAs in the gut.**

Lepr^db/db^ mice were supplemented with *A. muciniphila* for 8 weeks (-*AKK* and *+AKK*, respectively; n=6 per group), and the following analyses were performed: (A) PCoA plots showing unweighted UniFrac microbiota analysis; (B) Abundance of *Roseburia*, *Bifidobacterium*, *Turicibacter* and *Lachnospiraceae* at OUT level (n=6 per group); (C) Examination of short-chain fatty acids in feces (SCFAs; n=5-6 per group); (D) qRT-PCR analysis of mRNA levels *GPR43*, *GPR41*, *JNK1*, *JNK2*, *ERK2* and *P38* in inguinal white adipose tissue (n=3 per group); (E) Percentage of acetate, propanoic acid, isobutyric acid, butyrate, isovaleric acid and valeric acid in total SCFAs between eight groups (n=5-6 per group). Data are shown as means ± SEM. Statistical analyses were performed using Student’s t test; **P* < 0.05; ***P* < 0.01.

**Sup Figure 7. Effect of butyrate supplementation on high fat diet-fed mice.**

High fat diet-fed mice (HFD) were supplemented with 1% sodium butyrate (HFD+But) for 60 days, after which the following were analyzed: (A) Body weight gain, liver weight, and mass of gonadal white adipose tissue (gWAT) and inguinal white adipose tissue (iWAT) (n=6 per group); (B) Representative hematoxylin-eosin (H&E) staining of gWAT sections, Oil Red O staining of liver and muscle sections (n=3 per section per group); (C) The detection of triglyceride (TG) content in gWAT, liver and muscle (n=4 per group); (D) Representative H&E staining of interscapular fat sections (n=3 per section per group) and representative UCP1 immunohistochemical staining of interscapular fat and iWAT sections (n=3-4 per section per group); UCP1-positive signals were analyzed using *Image J software*; (E) Representative immunohistochemical staining of mitochondrial signaling (prohibitin) in iWAT, muscle and liver (n=3 per section per group); prohibitin-positive signals were analyzed using *Image J software*; (F) Glucose tolerance test (GTT, n=5 per group); (G) Insulin tolerance test (ITT, n=4-5 per group); (H) Rate of change in plasma glucose concentration versus time curve after performing ITT (n=4-5 per group); (I) Detection of serum levels of total serum TG (mmol/L), total cholesterol (TC, mmol/L), alanine aminotransferase (ALT, U/L), aspartate aminotransferase (AST, U/L), low-density-lipoprotein cholesterol (LDL, mmol/L), and high-density-lipoprotein cholesterol (HDL, mmol/L); (n=3-4 per group). Data are shown as means ± SEM. Statistical analyses were performed using Student’s t test; **P* < 0.05; ***P* < 0.01.

**Sup Figure 8. Effect of acetate supplementation on high fat diet-fed mice.**

HFD-fed mice were supplemented with 150 mM sodium acetate or without for 45 days (HFD+Ace and HFD, respectively), and the following were analyzed: (A) Weight gain (n=6 per group); (B) liver weight (n=6 per group); (C) Mass of gonadal white adipose tissue (gWAT, n=5-6 per group); (D) Mass of inguinal white adipose tissue (iWAT) (n=5 per group); (E) Detection of triglyceride (TG) levels in three metabolic tissues (n=3-4 per group); (F) Representative immunohistochemical staining of mitochondrial signaling (prohibitin) in histological sections of three metabolic tissues (n=3 per section per group); prohibitin-positive signals were analyzed using *Image J software*. Data are shown as means ± SEM. Statistical analyses were performed using Student’s t test; **P* < 0.05; ***P* < 0.01.

**Sup Figure 9. The effect of gut microbiome on Fgf21 of HFD-fed mice.**

qRT-PCR analysis of Fgf21 expression in liver or gonadal white adipose tissue after HFD-fed mice were treated with an antibiotic cocktail (Abx). Data are shown as means ± SEM. n=3 per group. Statistical analyses were performed using Student’s t test; **P* < 0.05; ***P* < 0.01.

**Fig. S10. Pathway and expression analysis of differently expressed miRNAs.**

(A) KEGG pathway enrichment analysis of targets of differentially expressed miRNAs; (B) Heat maps showing the expression levels (log10 [FPKM]) of miRNAs in inguinal white adipose tissue (iWAT) after various treatments. Data are shown as means ± SEM. n=3 per group. Statistical analyses were performed using Student’s t test (B); **P* < 0.05; ***P* < 0.01.

**Sup Figure 11. The effect of miR-378a family on obesity.**

High fat diet-fed mice (HFD) were injected intraperitoneally with a precursor of miR-378a-3p and miR-378a-5p (HFD+miR-378a) for 45 days, and the following were analyzed: (A) qRT-PCR analysis of mRNAs levels of miR-378a-3p and miR-378a-5p in liver and white adipose tissues (WAT, n=3 per group); (B) Representative hematoxylin-eosin (H&E) or EdU staining of gonadal white adipose tissues section (gWAT, n=3 per section per group); (C) Mass of interscapular fat (iBAT, n=5 per group); (D) Representative UCP1 immunohistochemical staining of interscapular fat section (n=3 per section per group); UCP1-positive signals were analyzed using *Image J software*; (E) Representative UCP1 immunohistochemical staining of gWAT and inguinal white adipose tissues section (iWAT, n=3 per section per group); UCP1-positive signals were analyzed using *Image J software*; (F) Glucose tolerance test (GTT, n=4-5 per group); (G) insulin tolerance test (ITT, n=4 per group); (H) Representative Oil Red O staining of liver sections (n=3 per section per group), and detection of triglyceride (TG) in liver (n=3-4 per group). Data are shown as means ± SEM. Statistical analyses were performed using Student’s t test; **P* < 0.05; ***P* < 0.01.

**Sup Figure 12.** **miR-378a-3p/5p are regulated by DNA methylation.**

(A) Location of CpG islands in the MIR-378a promoter; (B) Expression of miR-378a-3p/5p in 3T3-L1 cells treated with 4 μM 5-Aza-2 deoxycytidine (5-Aza-dC; n=3 per group) or without for 24 h; mouse 3T3-L1 cells were transfected with the siRNA of negative control (siNC), *Dnmt1* (siDnmt1), *Dnmt3a* (siDnmt3a) or *Dnmt3b* (siDnmt3b) for 48 h, and the following were analyzed: (C, D) mRNA levels of *Dnmt1*, *Dnmt3a*, *Dnmt3b, miR-378a-3p and miR-378a-5p* (n=3 per group)*;* (E) Quantification of methylation of the miR-378a promoter in iWAT and liver from betaine-related groups using a mass-spectrometry based method (n=3 per group). Data are shown as means ± SEM. Statistical analyses were performed using Student’s t test; **P* < 0.05; ***P* < 0.01.

**Sup Figure 13. *YY1* negatively correlates with miR-378a-3p and miR-378a-5p.**

(A) Sequence alignment of miR-378a-3p/5p with the 3'-UTR of *YY1*; the recombinant double-fluorescent reporter plasmid contains either a wild type (WT) or mutant (MUT) 3'-UTR of *YY1*. Red, seed sequence of miR-378a-3p/5p; Blue, wild-type and mutant seed regions; qRT-PCR analysis of *YY1* in (B) gonadal white adipose tissue (gWAT; n=4 per group), (C) inguinal white adipose tissue (iWAT; n=3 per group), (D) muscle (n=3 per group) and (E) liver (n=3 per group), after mice fed with high fat diet (HFD) and betaine (HFD+B) were treated with an antibiotic cocktail (HB+Abx); (F-J) qRT-PCR analysis of *YY1* mRNA levels in these three tissues in different treatment models (n=3-4 per group); Expression levels of (K) miR-378a-3p (n=3 per group), (L) miR-378a-5p (n=3 per group) and (M) *YY1* (n=3 per group) in mouse 3T3-L1 cells transfected with miR-378a-3p mimics (3p-Mimics), miR-378a-3p inhibitors (3p-Inhibitors), miR-378a-5p mimics (5p-Mimics), miR-378a-5p inhibitors (5p-Inhibitors) or a negative control (NC). Data are shown as means ± SEM. Statistical analyses were performed using Student’s t test; **P* < 0.05; ***P* < 0.01.
