# Supplementary figures and images for: Dietary betaine prevents obesity through gut microbiota-drived microRNA-378a family
Source: Gut Microbes. 2021 Feb 8;13(1):1862612. doi: 10.1080/19490976.2020.1862612 (PMC7889173; doi:10.1080/19490976.2020.1862612)

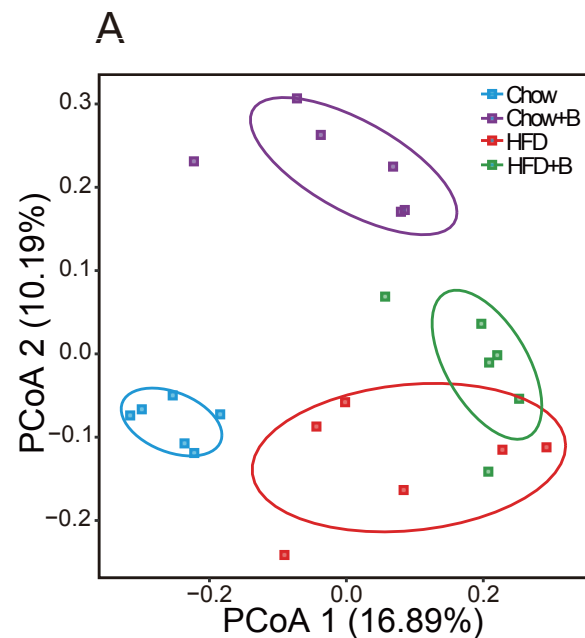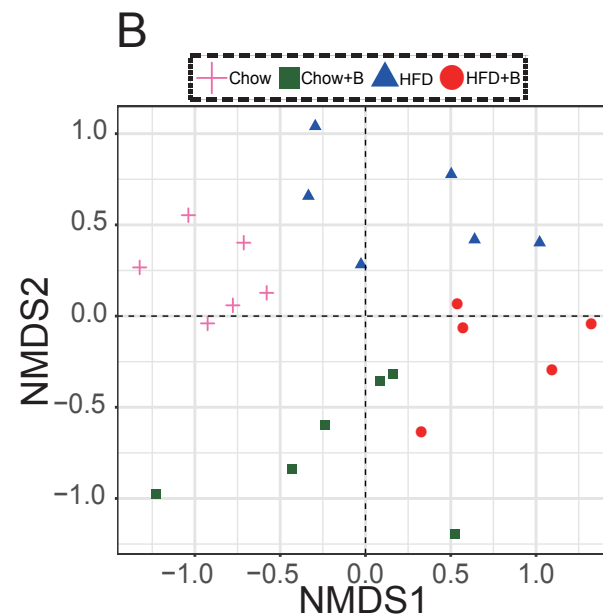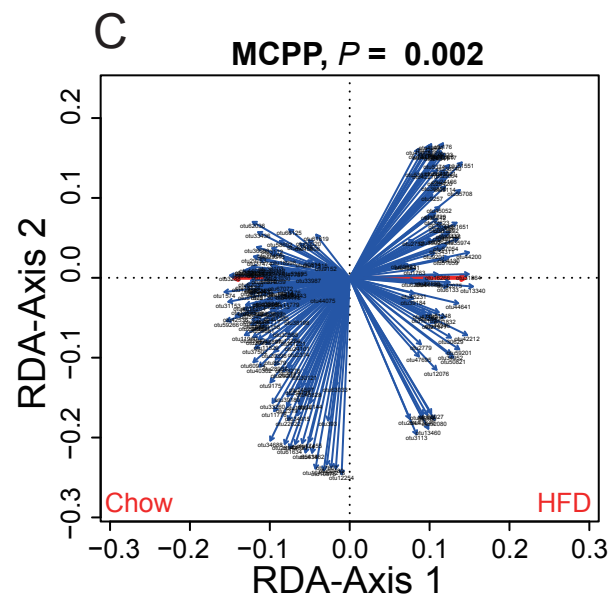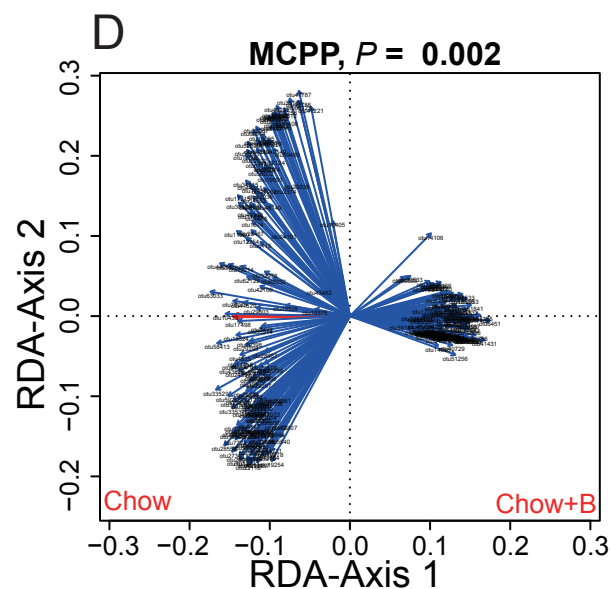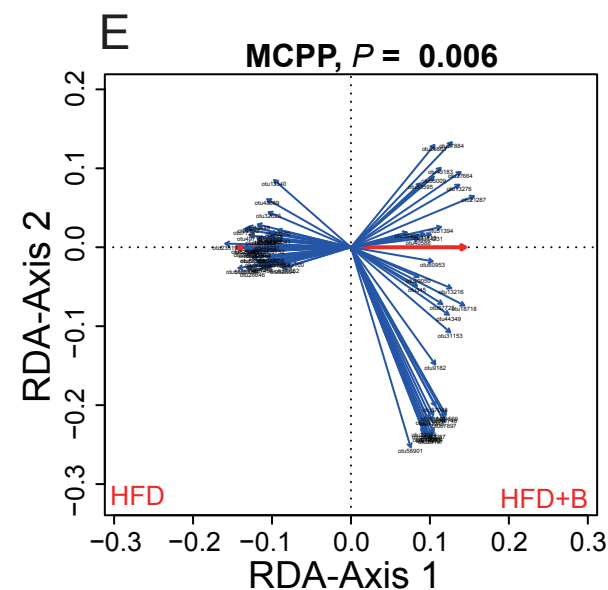

Supplement: Supplemental Material [file KGMI_A_1862612_SM6571.zip › supplementary/Fig.S1.pdf]

A

KEGG Pathway Enrichment list

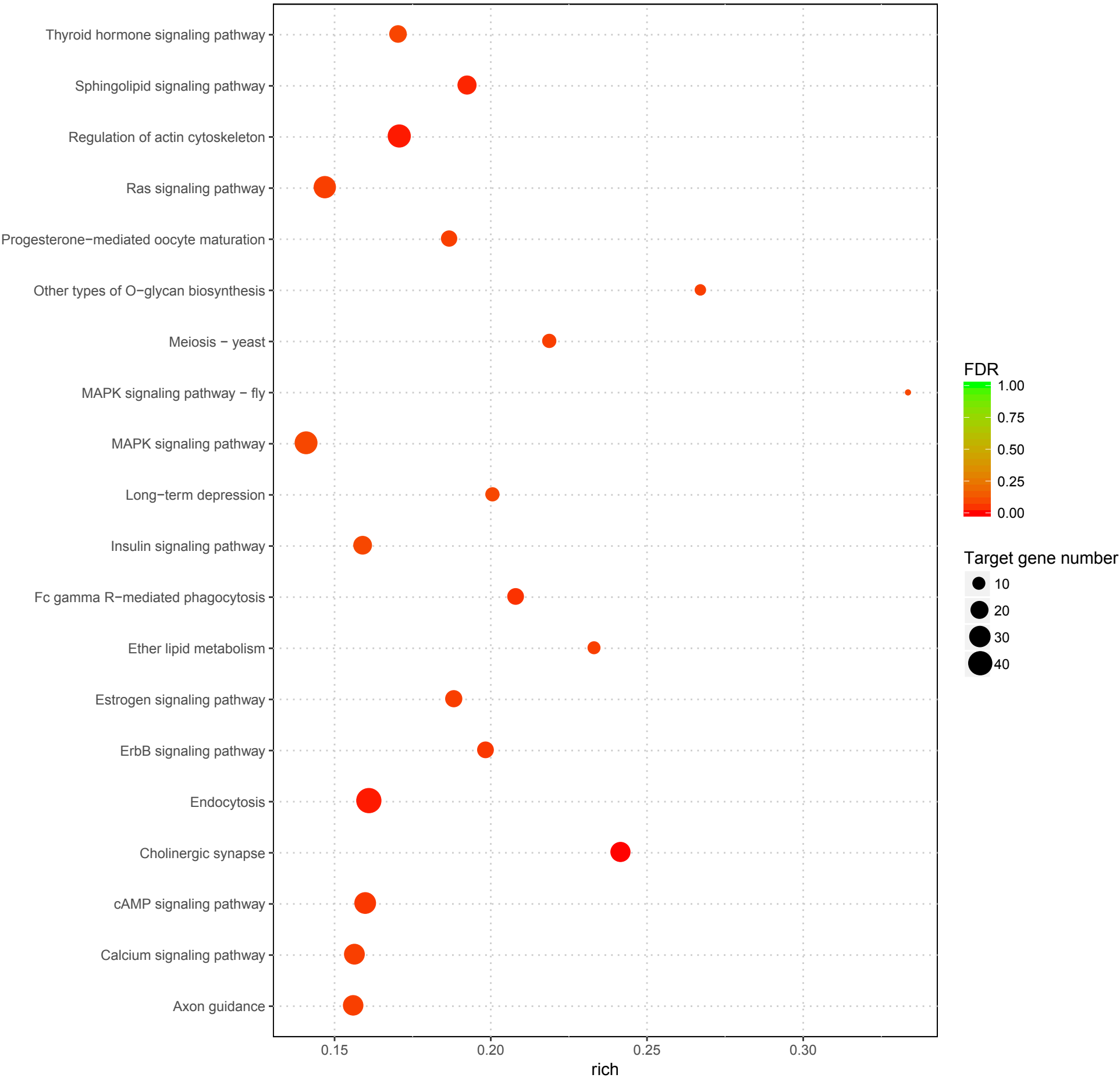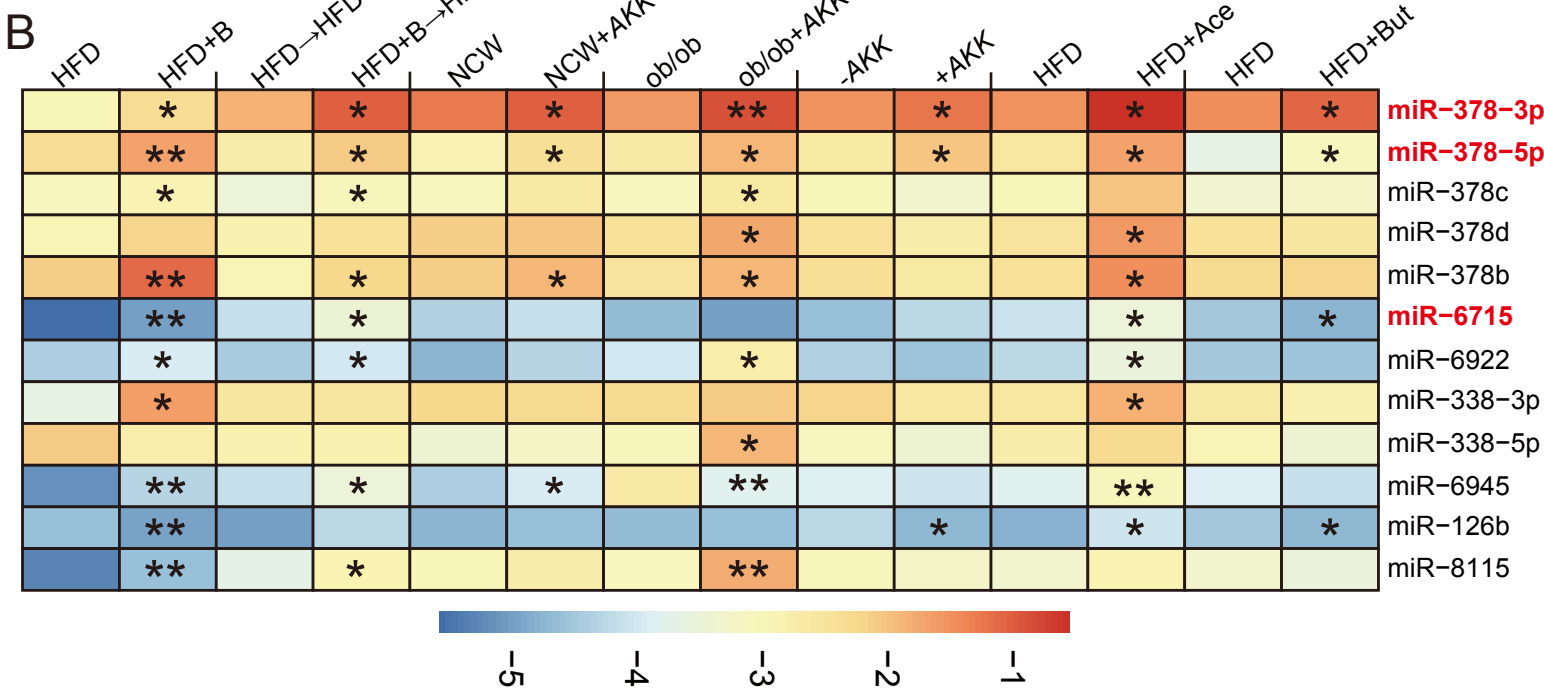

Supplement: Supplemental Material [file KGMI_A_1862612_SM6571.zip › supplementary/Fig.S10.pdf]

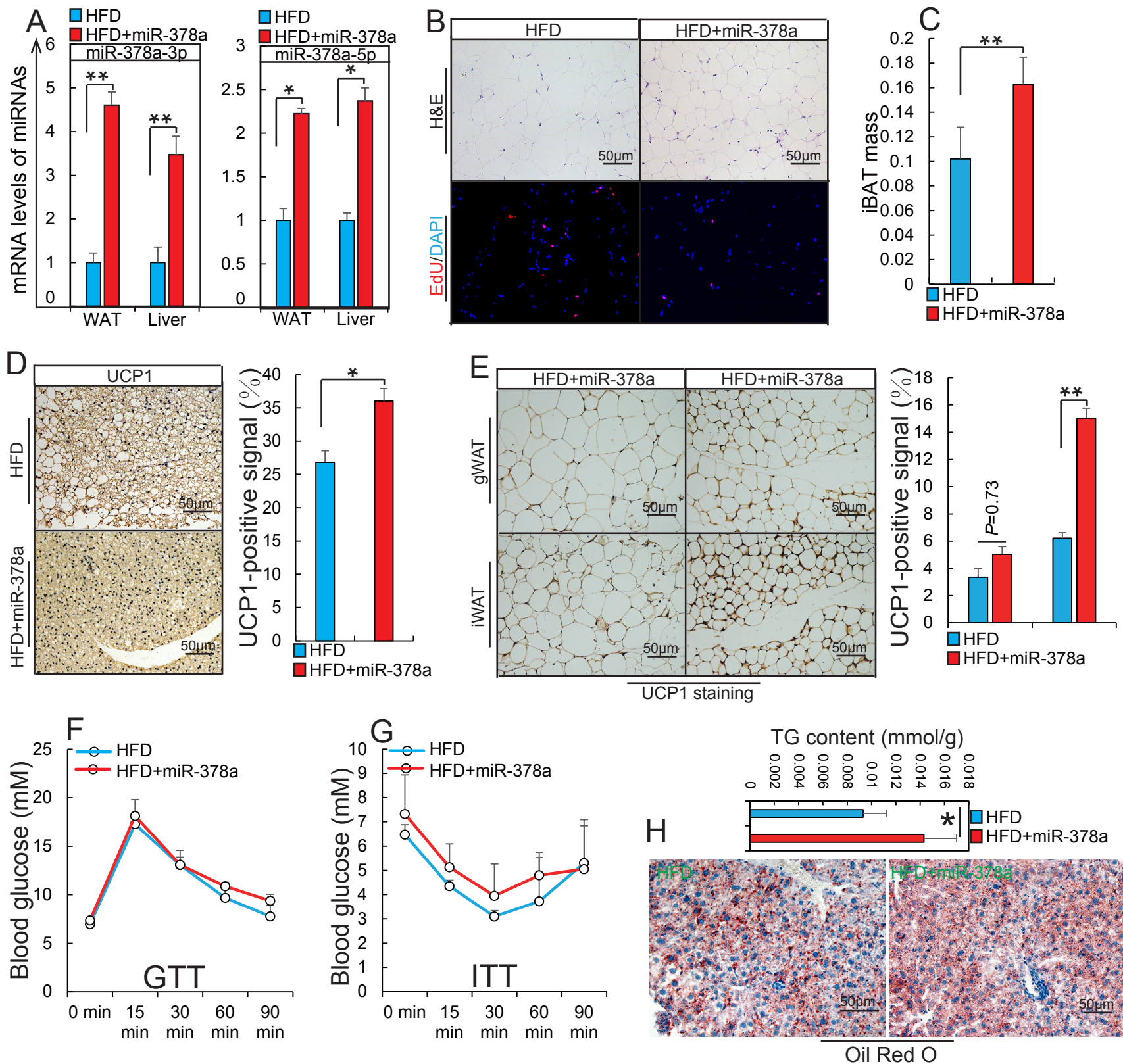

Supplement: Supplemental Material [file KGMI_A_1862612_SM6571.zip › supplementary/Fig.S11.pdf]

A

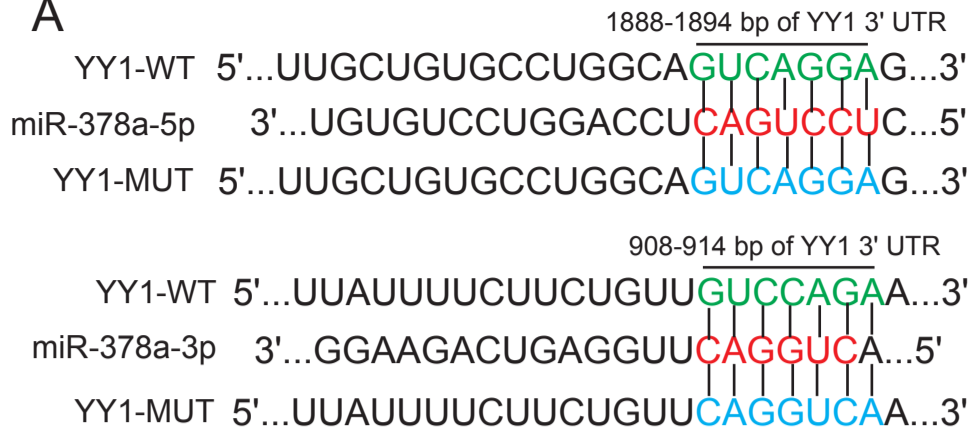

B

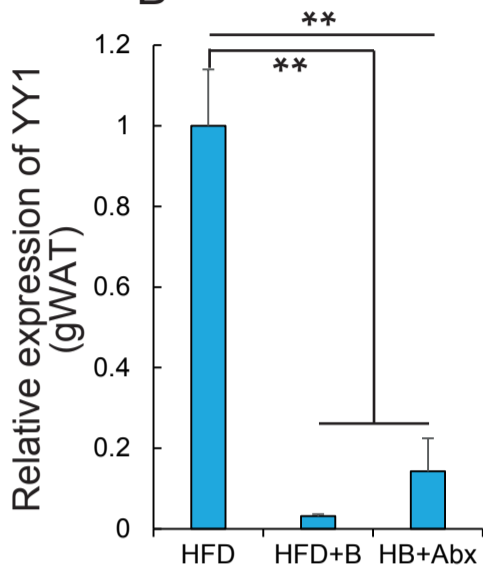

C

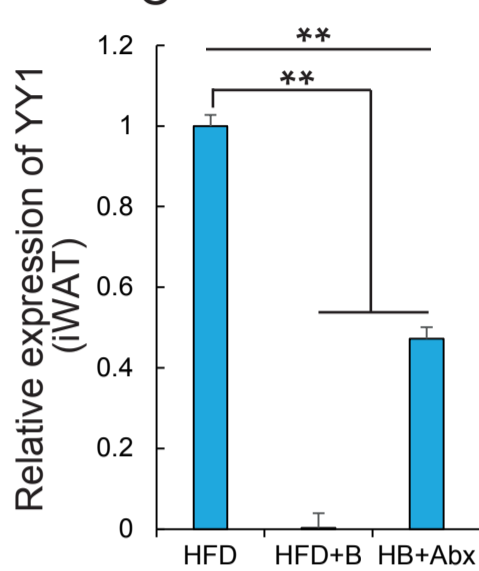

D

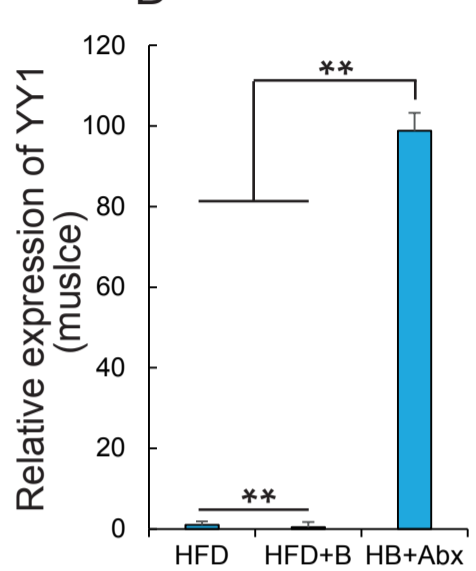

E

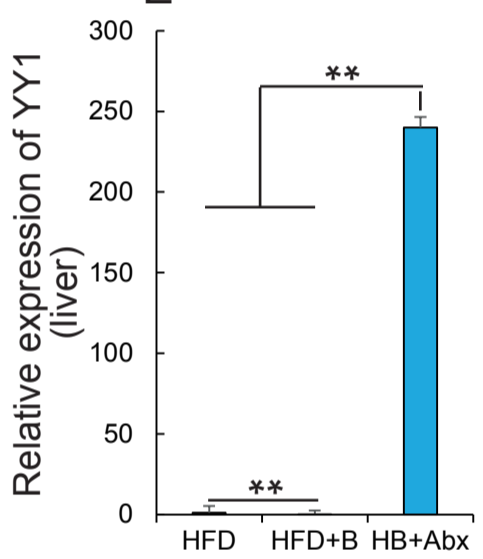

F

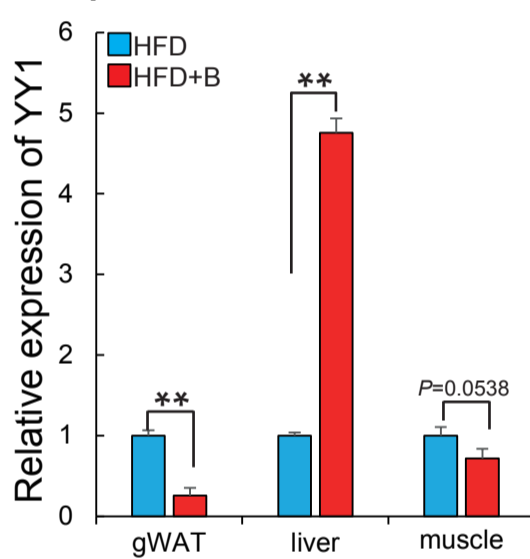

G

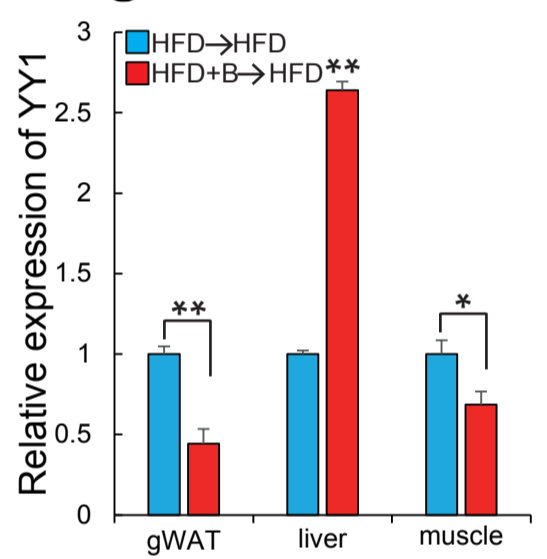

H

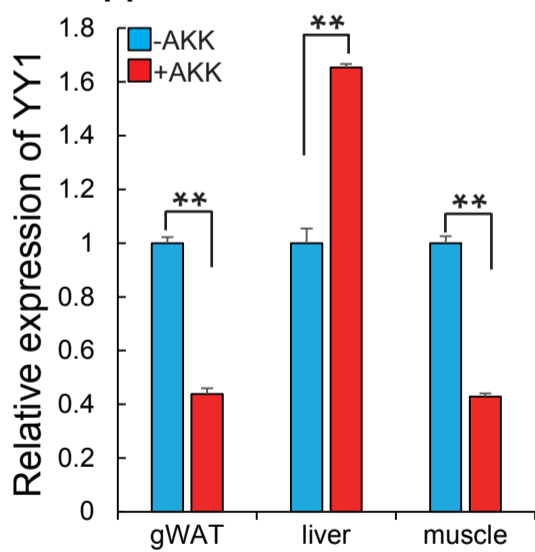

I

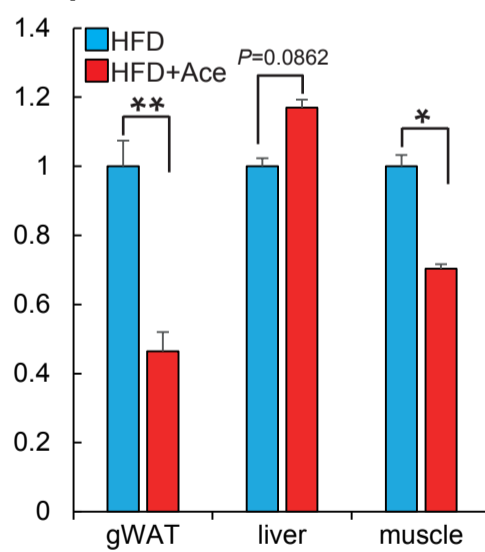

J

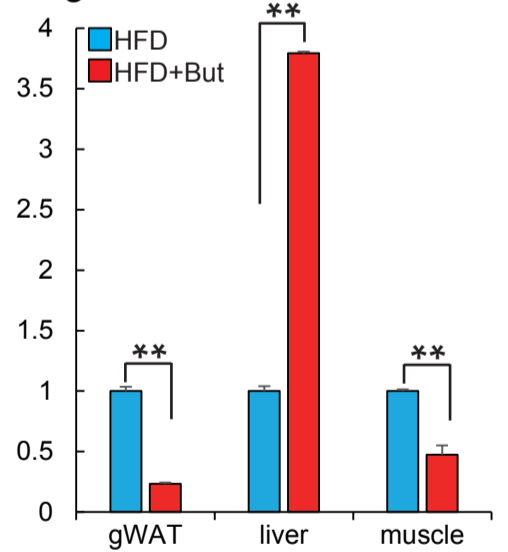

K

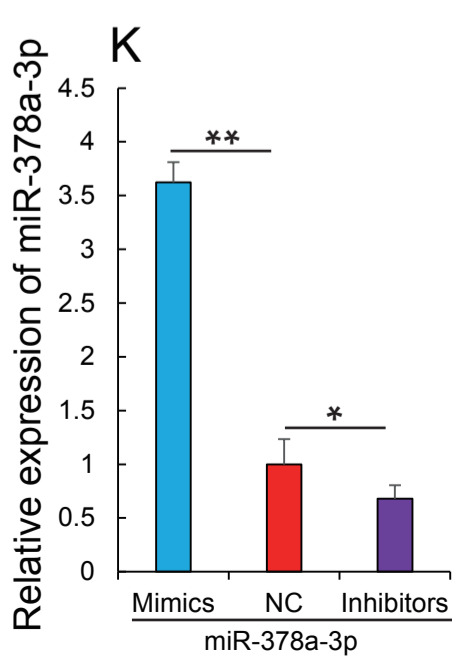

L

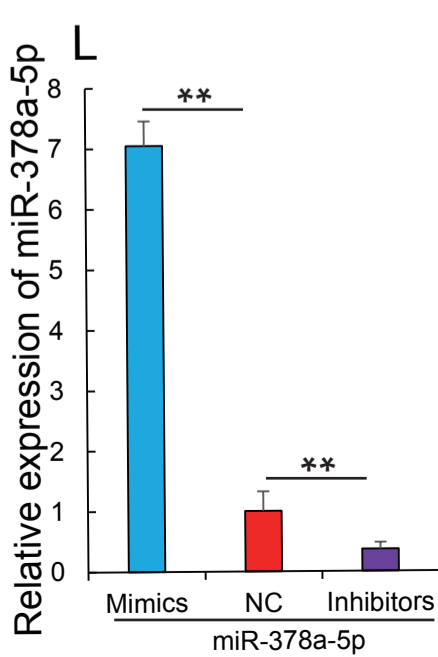

M

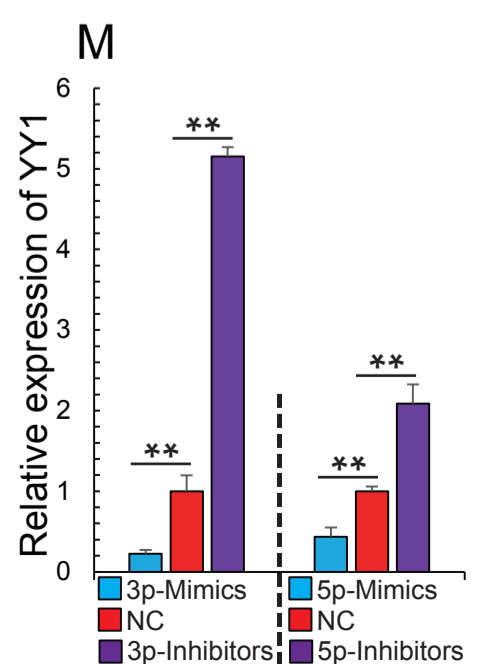

Supplement: Supplemental Material [file KGMI_A_1862612_SM6571.zip › supplementary/Fig.S13.pdf]

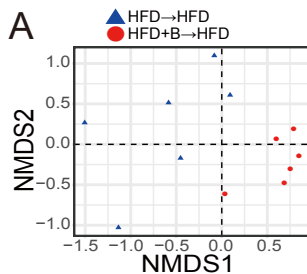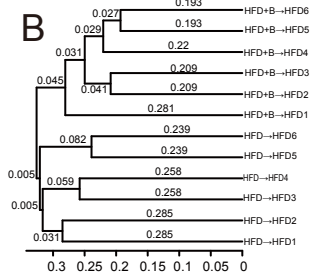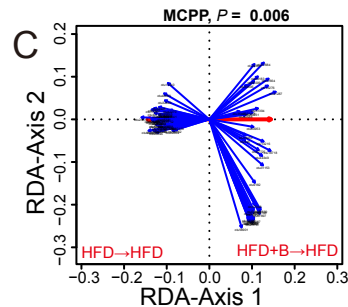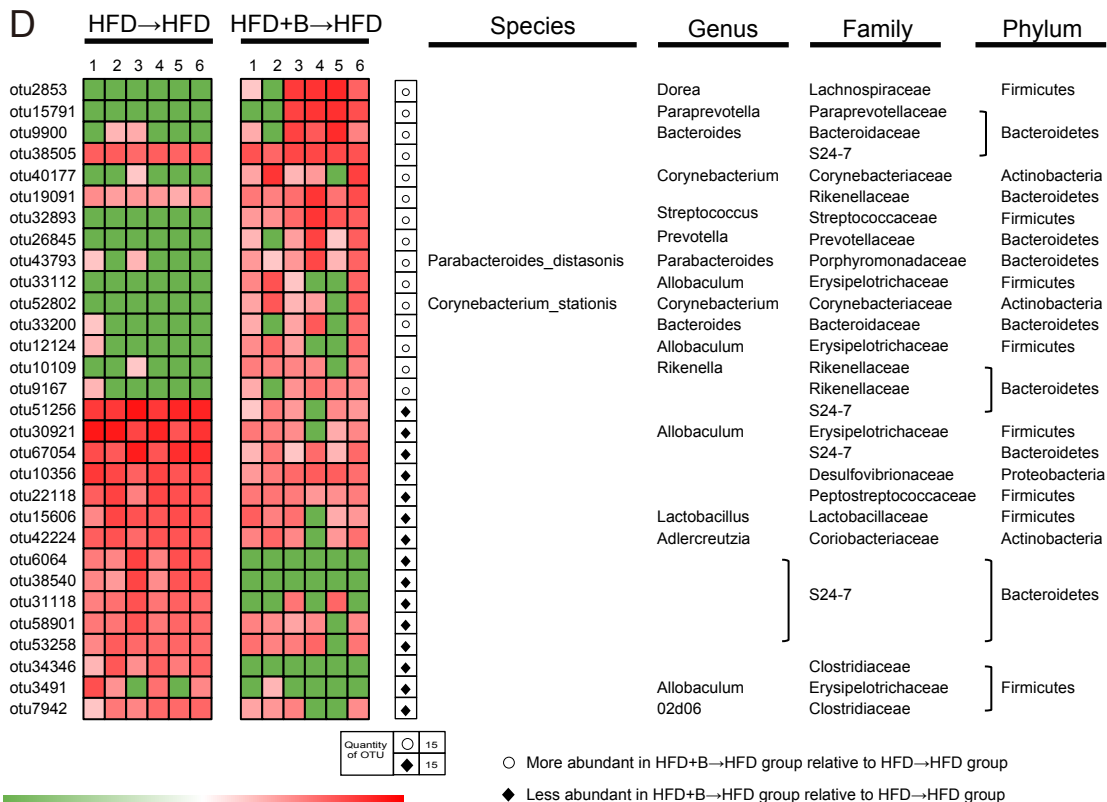

0.0 0.001 30

Supplement: Supplemental Material [file KGMI_A_1862612_SM6571.zip › supplementary/Fig.S2.pdf]

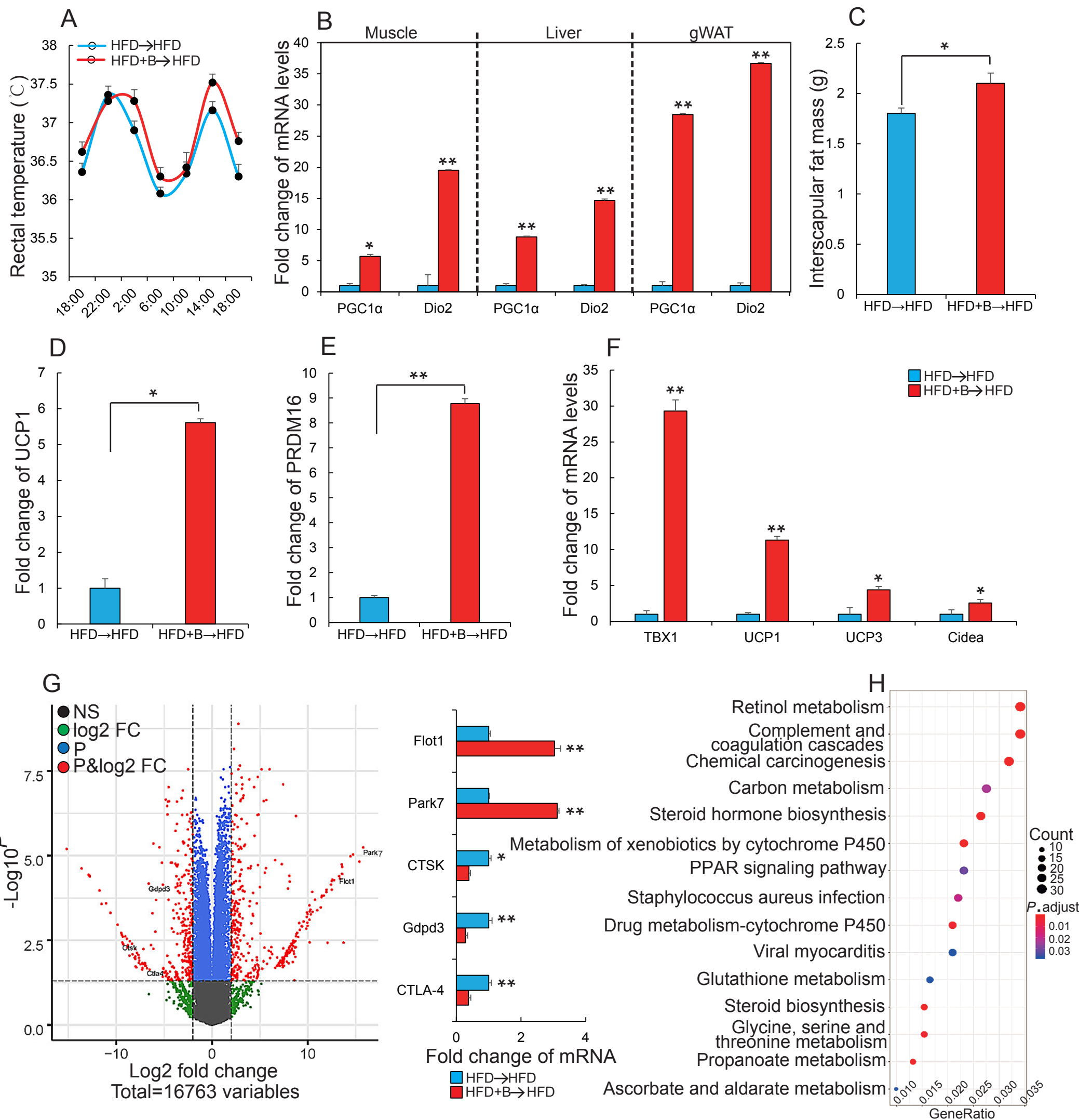

Supplement: Supplemental Material [file KGMI_A_1862612_SM6571.zip › supplementary/Fig.S3.pdf]

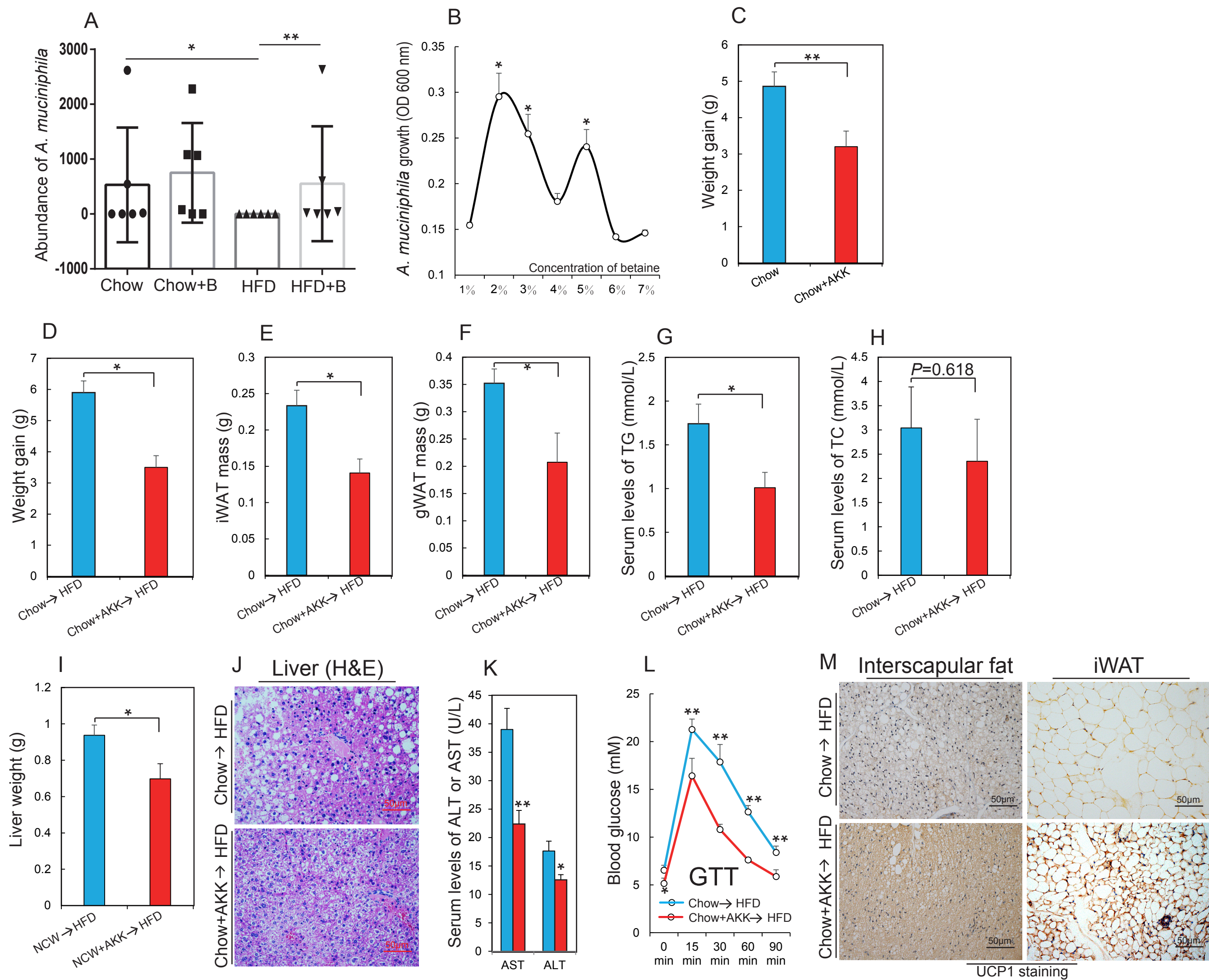

Supplement: Supplemental Material [file KGMI_A_1862612_SM6571.zip › supplementary/Fig.S4.pdf]

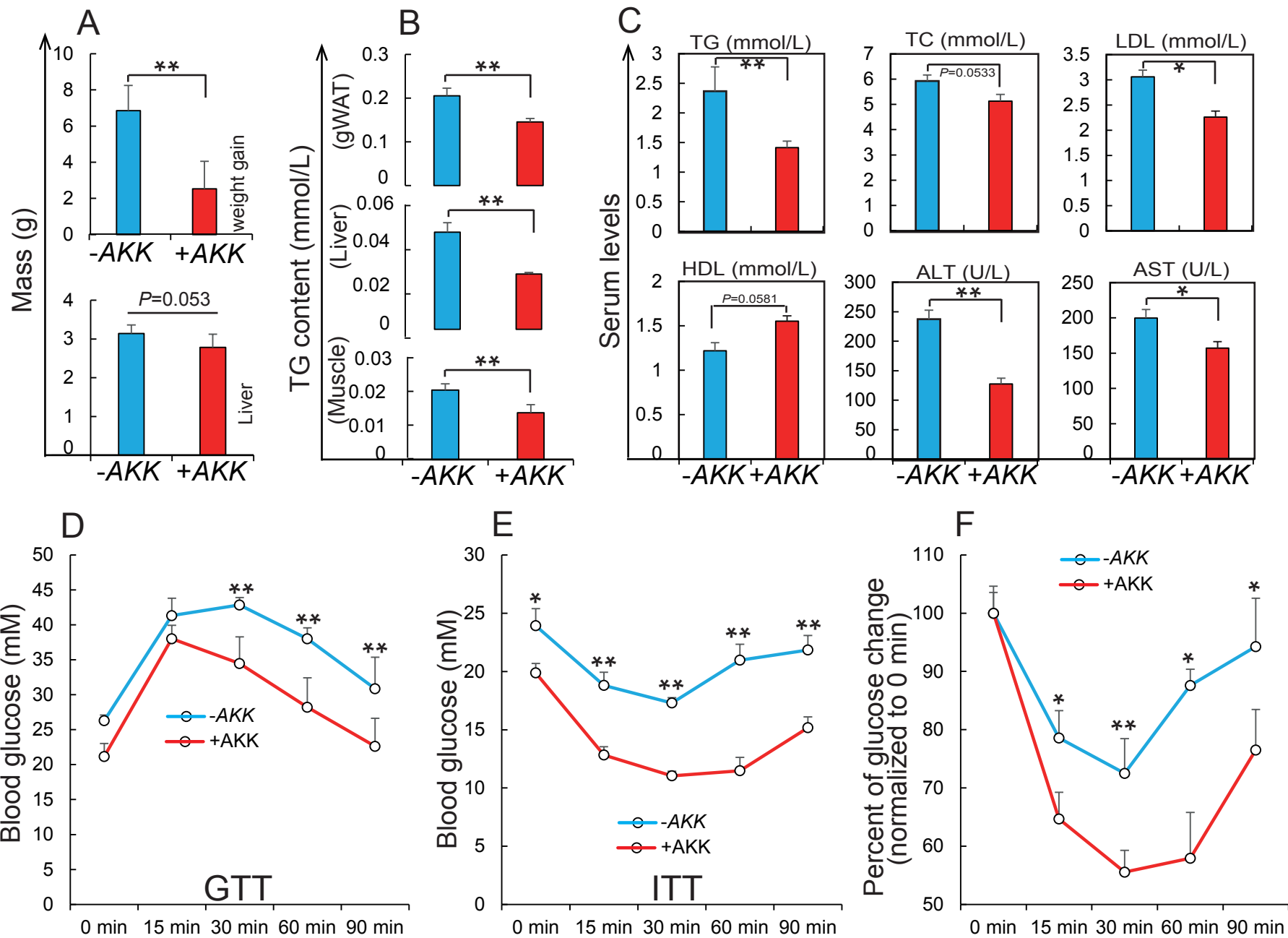

Supplement: Supplemental Material [file KGMI_A_1862612_SM6571.zip › supplementary/Fig.S5.pdf]

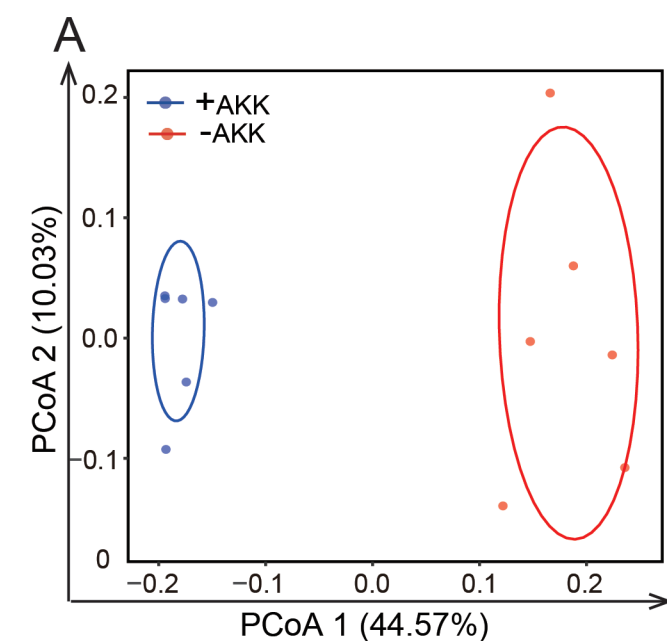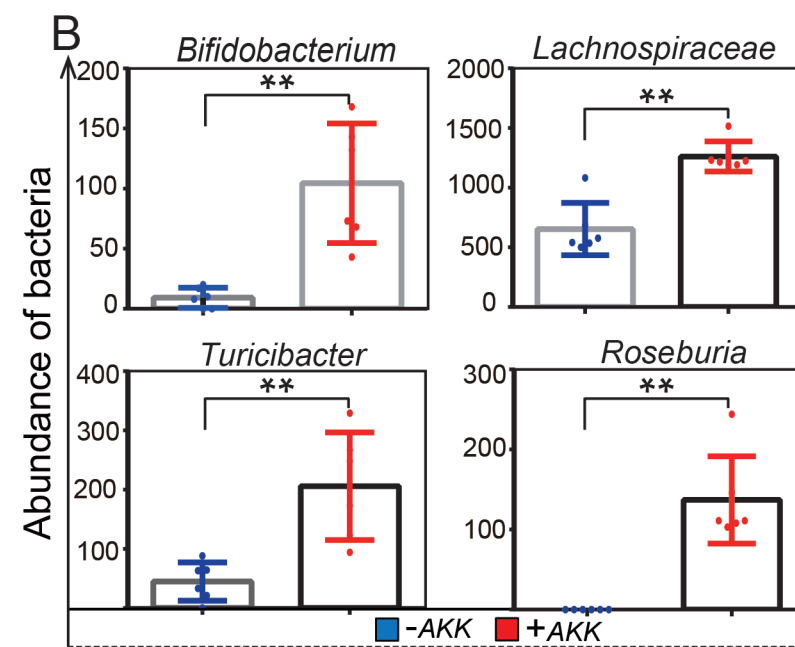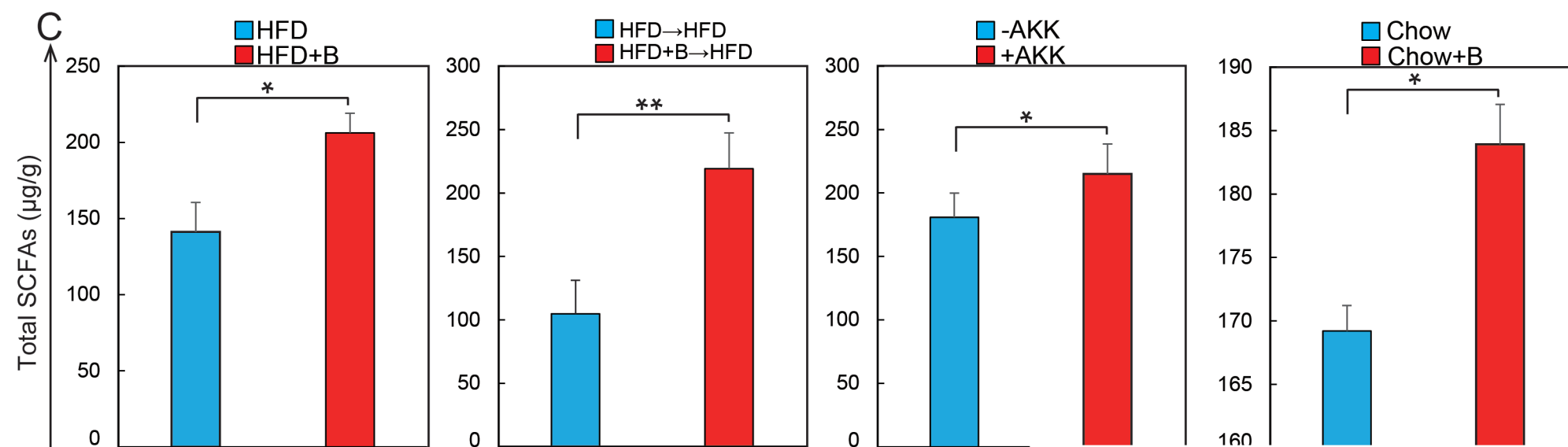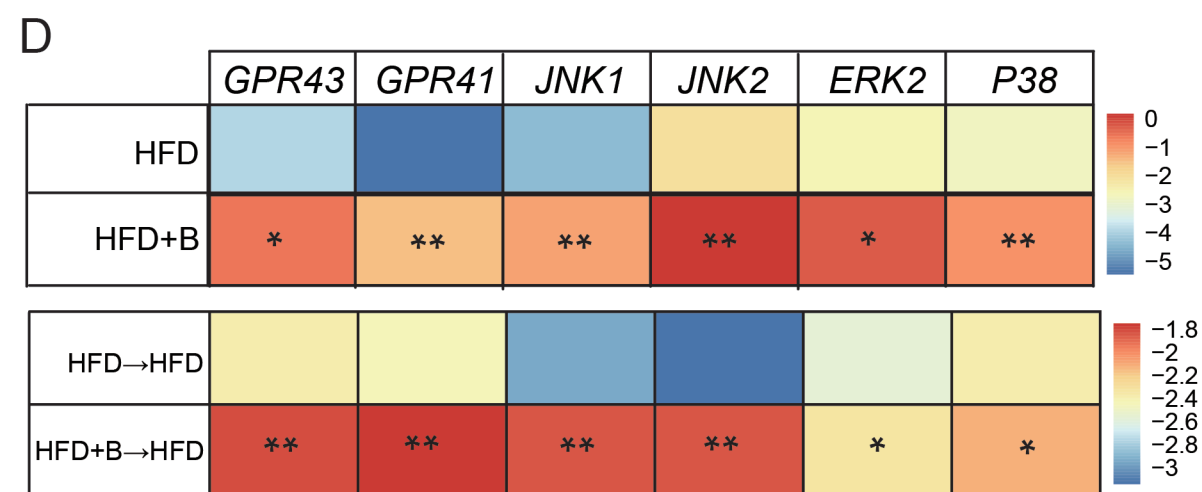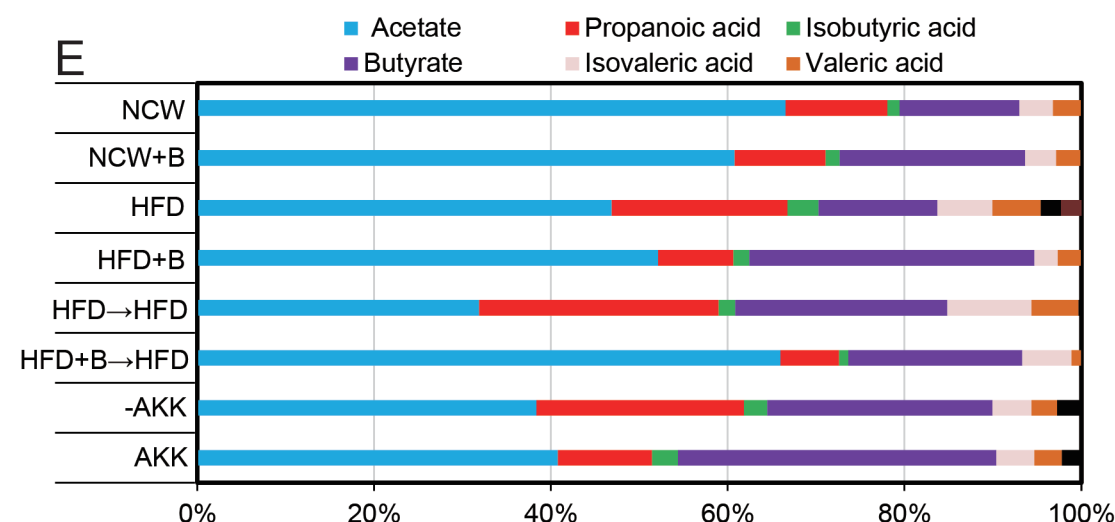

Supplement: Supplemental Material [file KGMI_A_1862612_SM6571.zip › supplementary/Fig.S6.pdf]

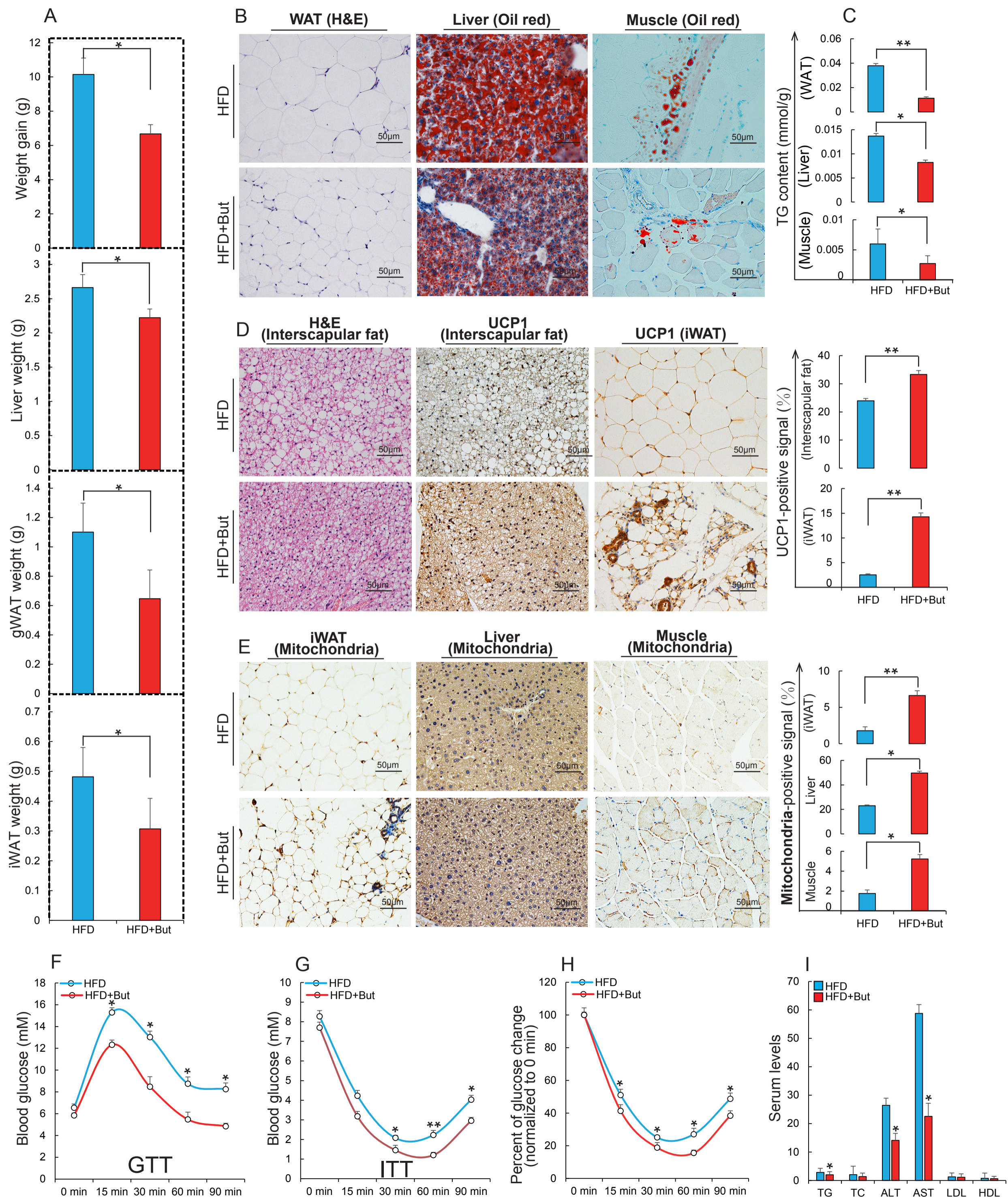

Supplement: Supplemental Material [file KGMI_A_1862612_SM6571.zip › supplementary/Fig.S7.pdf]

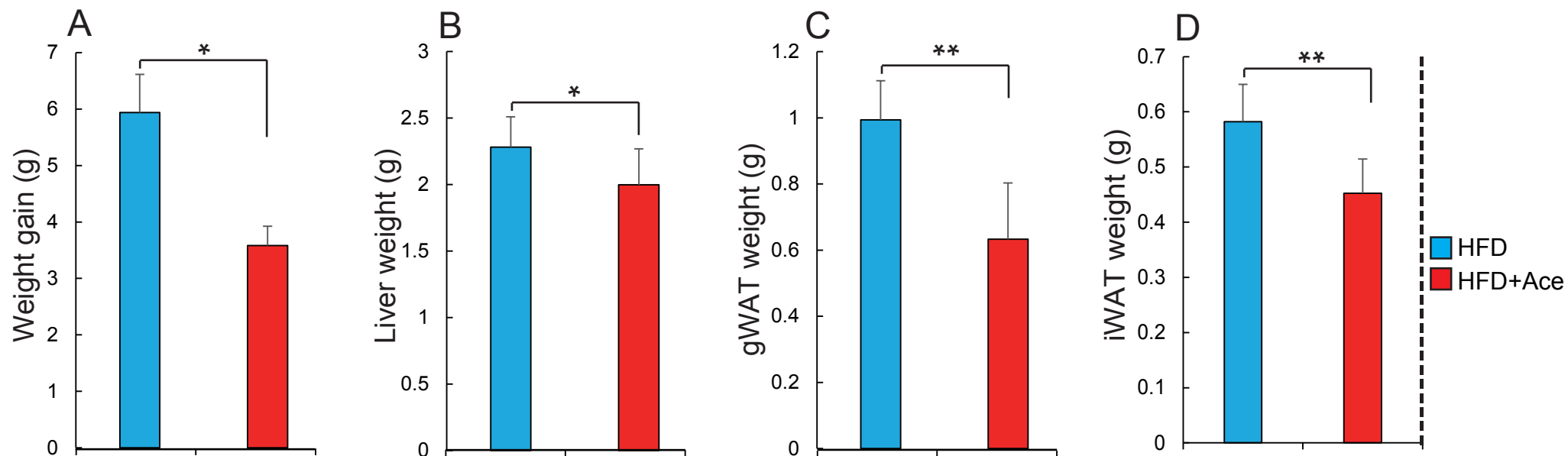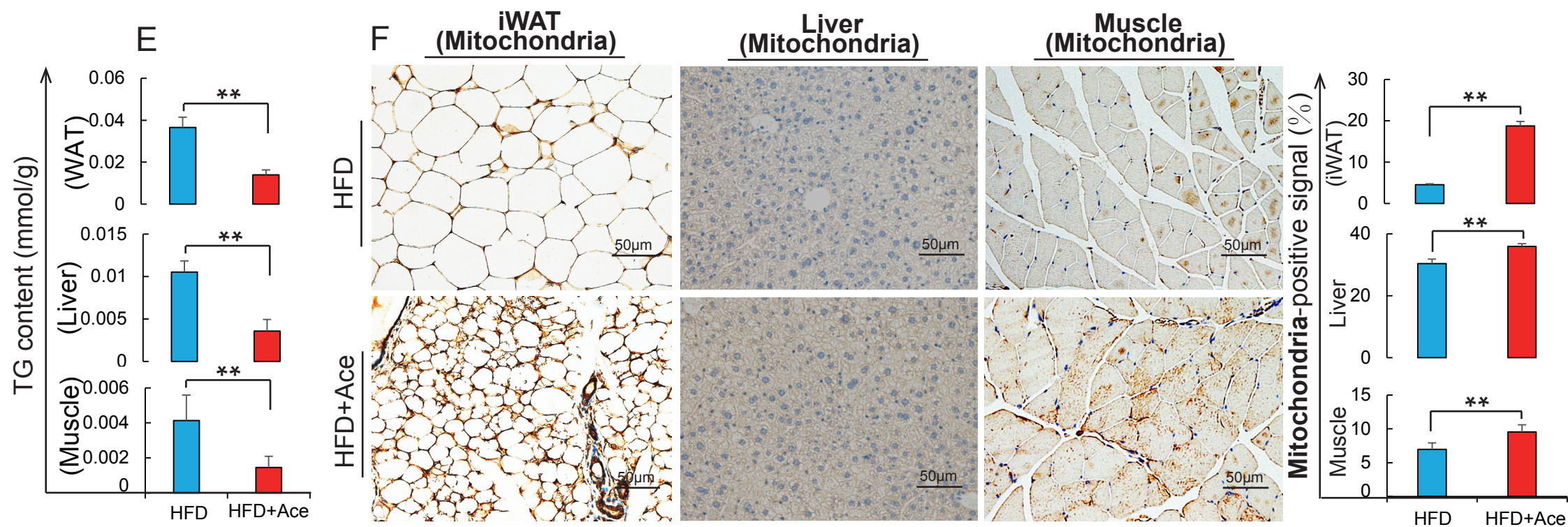

Supplement: Supplemental Material [file KGMI_A_1862612_SM6571.zip › supplementary/Fig.S8.pdf]

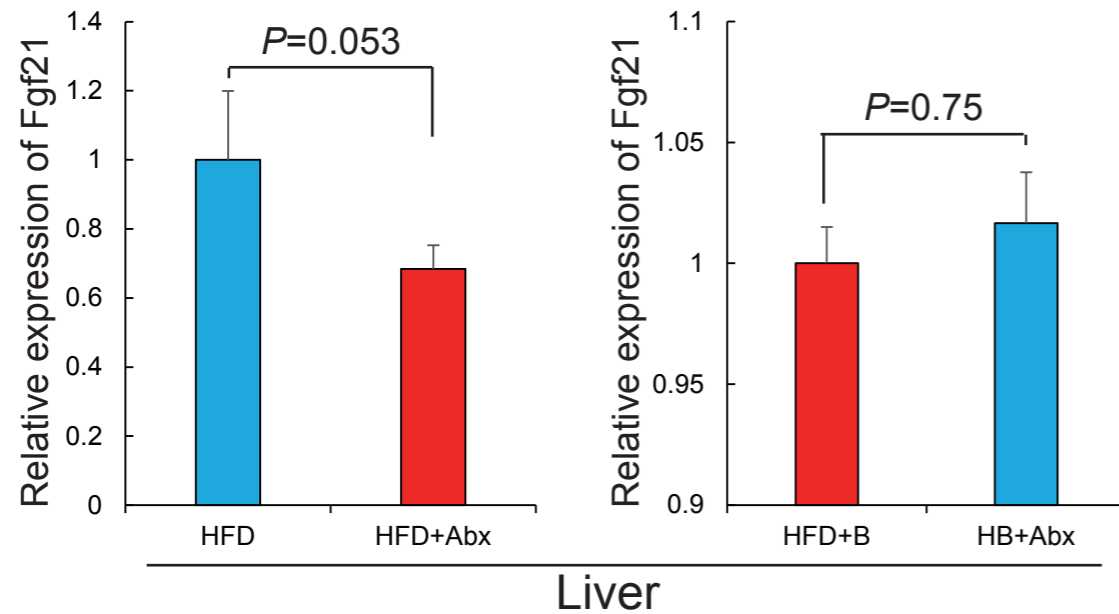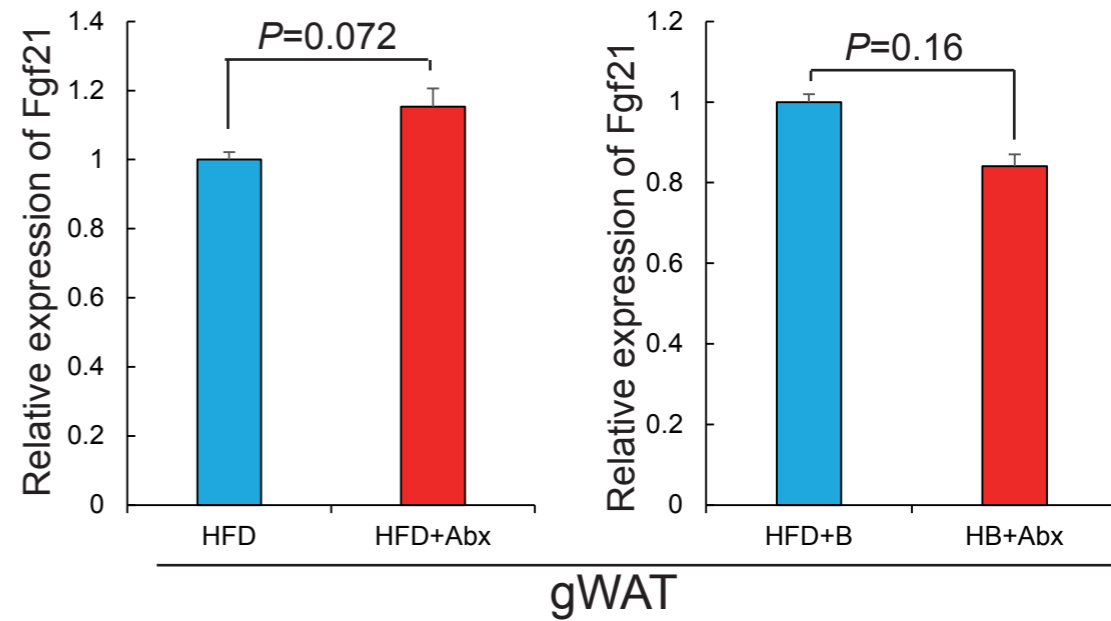

Supplement: Supplemental Material [file KGMI_A_1862612_SM6571.zip › supplementary/Fig.S9.pdf]
