# Supplementary material for: Dietary betaine prevents obesity through gut microbiota-drived microRNA-378a family
Source: Gut Microbes. 2021 Feb 8;13(1):1862612. doi: 10.1080/19490976.2020.1862612 (PMC7889173; doi:10.1080/19490976.2020.1862612)
Supplement: Supplemental Material [file Fig.S12.pdf › supplementary/Fig.S12.pdf]

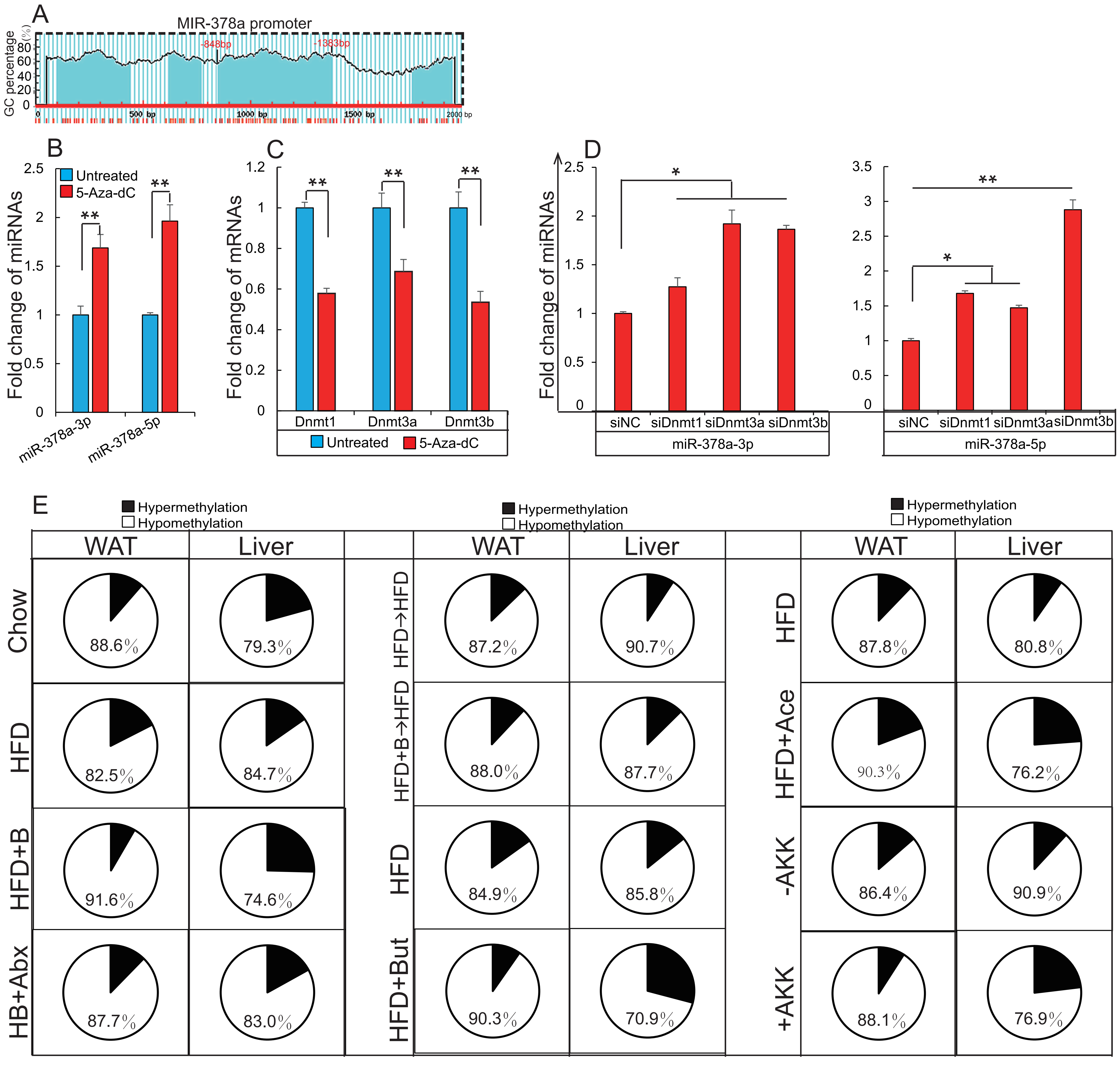
A
MIR-378a promoter
GC percentage (%)
-848bp
-1383bp
500 bp
1000 bp
1500 bp
2000 bp
B
Untreated
5-Aza-dC
Fold change of miRNAs
miR-378a-3p
miR-378a-5p
C
Fold change of mRNAs
Dnmt1
Dnmt3a
Dnmt3b
Untreated
5-Aza-dC
D
Fold change of miRNAs
siNC
siDnmt1
siDnmt3a
siDnmt3b
miR-378a-3p
siNC
siDnmt1
siDnmt3a
siDnmt3b
miR-378a-5p
E
Hypermethylation
Hypomethylation
WAT
Liver
Chow
88.6%
79.3%
HFD
82.5%
84.7%
HFD+B
91.6%
74.6%
HB+Abx
87.7%
83.0%
HFD→HFD
87.2%
90.7%
HFD+B→HFD
88.0%
87.7%
HFD
84.9%
85.8%
HFD+But
90.3%
70.9%
HFD
87.8%
80.8%
HFD+Ace
90.3%
76.2%
-AKK
86.4%
90.9%
+AKK
88.1%
76.9%
